# Hot exciplexes in U-shaped TADF molecules with emission from locally excited states

**DOI:** 10.1038/s41467-021-26439-w

**Published:** 2021-10-26

**Authors:** A. Lennart Schleper, Kenichi Goushi, Christoph Bannwarth, Bastian Haehnle, Philipp J. Welscher, Chihaya Adachi, Alexander J. C. Kuehne

**Affiliations:** 1 Institute of Organic and Macromolecular Chemistry, Ulm University, Albert-Einstein-Allee 11, 89081 Ulm, Germany; 2 Department of Applied Chemistry and Center for Organic Photonics and Electronics Research (OPERA), Kyushu University, 744 Motooka, Nishi, Fukuoka 819-0395 Japan; 3 International Institute for Carbon Neutral Energy Research (WPI-I2CNER), Kyushu University, 744 Motooka, Nishiku, Fukuoka 819-0395 Japan; 4 Institute of Physical Chemistry, RWTH Aachen University, Landoltweg 2, 52074 Aachen, Germany; 5 DWI – Leibniz-Institute for Interactive Materials, Forckenbeckstraße 50, 52074 Aachen, Germany

**Keywords:** Optical materials, Single-molecule fluorescence, Organic LEDs

## Abstract

Fast emission and high color purity are essential characteristics of modern opto-electronic devices, such as organic light emitting diodes (OLEDs). These properties are currently not met by the latest generation of thermally activated delayed fluorescence (TADF) emitters. Here, we present an approach, called “hot exciplexes” that enables access to both attributes at the same time. Hot exciplexes are produced by coupling facing donor and acceptor moieties to an anthracene bridge, yielding an exciplex with large T_1_ to T_2_ spacing. The hot exciplex model is investigated using optical spectroscopy and quantum chemical simulations. Reverse intersystem crossing is found to occur preferentially from the T_3_ to the S_1_ state within only a few nanoseconds. Application and practicality of the model are shown by fabrication of organic light-emitting diodes with up to 32 % hot exciplex contribution and low efficiency roll-off.

## Abstract (web summary)

Molecular designs improving the performance of thermally activated delayed fluorescence (TADF) emitters are greatly desired for optoelectronic applications. The authors propose a molecular geometry with donor and acceptor moieties facing each other separated by an anthracene bridge, giving rise to hot exciplex states producing color pure and fast TADF emission.

## Introduction

**Fig. 1:**
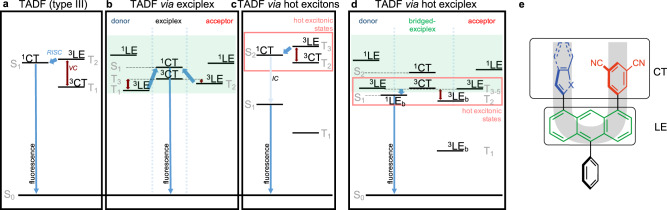
Design considerations for hot exciplex TADF emitters. **a** Type III TADF, where T_1_ and T_2_ are vibronically coupled (VC, dark red arrows) and RISC (bent blue arrows) occurs from T_2_ to S_1_, which is a CT. **b** In an exciplex, two charge transfer states are generated, the ^3^CT is vibronically coupled to locally excited triplet states on the donor and acceptor moieties and after RISC, emission occurs from the charge transfer singlet state ^1^CT. **c** In hot exciton TADF emitters, there is a large energy gap between T_1_ and T_2_ rendering the T_2_ stable, so that VC and RISC takes place between the hot triplet states and a singlet state. If that singlet is an upper excited state, internal conversion (IC) to the S_1_ can take place. Molecular design can render this state a locally excited state. **d** By bridging donor and acceptor with anthracene, a mixture of the exciplex and hot exciton mechanisms can be produced. Here, hot excitonic states are produced with participation by the exciplex states. As a result, the S_1_ is always a ^1^LE state, which is beneficial for high-efficiency emitters. The subscript b indicates that this state is localized on the anthracene bridge. **e** U-shaped hot exciton design, containing an anthracene unit (green) which holds the LE and bridges the donors (blue; X = O, S) and acceptor (red) moieties. The acceptor unit in all cases is isophtalonitrile; employed donor units are methylfuran, benzofuran, methylthiophene, and benzothiophene. The phenyl ring (black) improves solubility.

Thermally activated delayed fluorescence (TADF) has emerged as a powerful approach to improve the performance of optoelectronic devices by converting passive triplet excitons into fluorescent singlet excitons^1–5^. This way, the internal device efficiency can be improved theoretically from 25% to 100%, by harvesting virtually all generated excitons. Conventional TADF luminophores make use of geometrical confinement of the HOMO and LUMO, and precise interplay of electron donor and acceptor moieties. This geometry evokes a charge transfer type excited state (CT) allowing a small energy gap ∆*E*_ST_ between the lowest excited singlet state (S_1_) and the lowest excited triplet state (T_1_). The small ∆*E*_ST_ allows reverse intersystem crossing (RISC) in combination with vibronic coupling from T_1_ to S_1_ and fluorescence from there (see Fig. 1a). Following this approach, TADF-based OLEDs with internal quantum efficiencies of ~100% and high external quantum efficiencies (EQEs) of ~40% could be obtained^6–8^. Alternatively, charge transfer can also occur in exciplexes formed by individual electron donor and acceptor molecules. While both donor and acceptor possess locally excited singlet states (^1^LE), the emission occurs from the generated exciplex charge transfer singlet state ^1^CT (see Fig. 1b)^9^. Connecting donor and acceptor by a molecular bridge increases the yield of exciplex formation^10,11^. However, the involvement of a CT as the emissive state entails low oscillator strengths *f* and therefore inferior radiative relaxation efficiency and poor color purity. One strategy to overcome this problem is via hot triplet excitons that enable upper-level RISC, evading limitation to emissive ^1^CT states of conventional TADF materials (see Fig. 1c)^12–14^. These emitters often incorporate anthracene with its low lying T_1_ state, making it an obvious candidate for hot exciton TADF^15–19^. However, bilateral functionalization with electron donor and acceptor groups on opposite sides of the anthracene core leads to a mixture of LE and CT states, generating hybridized local and charge transfer states (HLCT)^19–23^. Unfortunately, the emission characteristics of such HLCT geometries exhibit strong dependence on the polarity of the environment, thus limiting the choice of matrix and the OLED device architecture^24–26^. Moreover, hot exciton luminophores with HLCT states show detrimental roll-off behavior in current efficiency and low EQE^27–29^.

New molecular designs that make use of precisely localized ^1^LE states for emission, while utilizing hot excitonic states for fast transfer to these ^1^LE states, would advance the field and understanding of TADF and enable the development of emissive materials with superior device performance in view of efficiency and color purity. Energetic alignment of these emissive ^1^LE states with ^3^CT states should boost RISC rates, leading to increased long-term stability and low efficiency roll-off^30,31^.

Here, we present and characterize a new class of TADF materials, where we achieve precise matching of LE to CT states. To accomplish this, we utilize a bridged exciplex geometry, which aligns donor and acceptor moieties maximizing the yield of contributing CT states. As bridge, we employ the anthracene motif, which gives access to hot exciton states and its innate fast upper-level RISC pathway and a ^1^LE singlet state for emission (see Fig. 1d). This new “hot exciplex” design suppresses HLCT behavior, even in strongly polar environment, allowing high RISC rates and emission with high oscillator strength *f* from the ^1^LE state. Emission from this ^1^LE state leads to short fluorescence lifetimes and high color purity in the blue spectrum^24,32^. We compare four different donor substituents and characterize our molecules by density functional theory (DFT) simulations and transient spectroscopy.

## Results and discussion

### Molecular design

In our molecular design, we aim at aligning CT and LE states to enable spin–orbit coupling between excited triplet and excited singlet states and to promote fast RISC^33–35^. However, CT and LE must not mix in such a way that they form HLCT states. To avoid HLCT formation, we devise a molecular architecture by unilateral functionalization of anthracene with electron donor and acceptor units (U-shaped, as depicted in Fig. 1e). This U-shaped molecular design aligns donor and acceptor to enable through-space charge transfer, like in an exciplex. The non-polar anthracene bridge that accommodates LE states connects donor and acceptor units hosting the CT states (see Fig. 1d, e). This molecular design confines CT and LE states to separate hemispheres of the molecule, thus avoiding mixing of CT with LE and HLCT behavior. As an acceptor we opt for isophthalonitrile, which has demonstrated excellent performance in various TADF active materials^3,4^. We compare four different donor units, namely methylfuran (MeFuPAI), benzofuran (BeFuPAI), methylthiophene (MeThPAI), and benzothiophene (BeThPAI). The opposite anthracene hemisphere is decorated with a phenyl ring, which is twisted with respect to the anthracene, to increase solubility and obviate crystallization.

### Synthesis

**Fig. 2:**
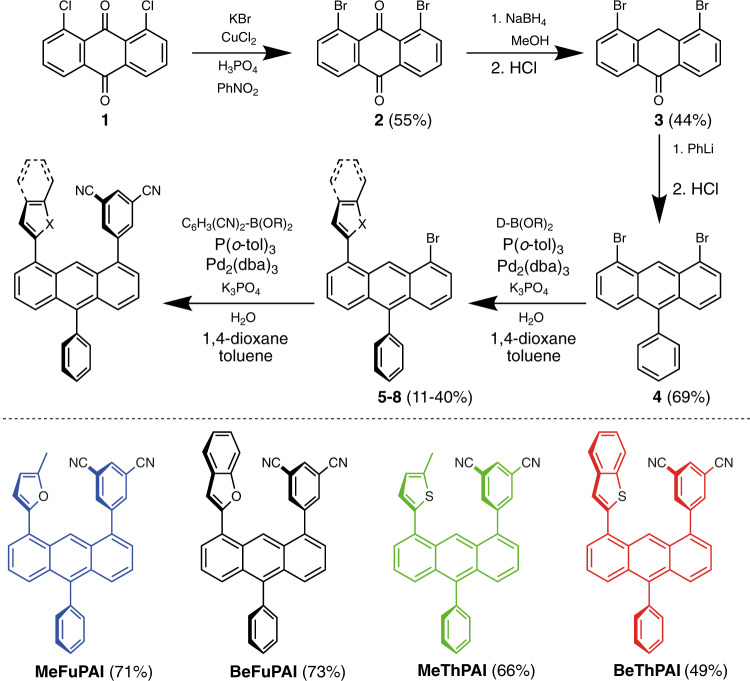
Synthetic route yielding the hot exciplex emitters. 5-(8-(5-Methylfuran-2-yl)-10-phenylanthracen-1-yl)isophthalonitrile (MeFuPAI), 5-(8-(benzofuran-2-yl)-10-phenylanthracen-1-yl)isophthalonitrile (BeFuPAI), 5-(8-(5-methylthiophen-2-yl)-10-phenylanthracen-1-yl)isophthalonitrile (MeThPAI), and 5-(8-(benzo[b]thiophen-2-yl)-10-phenyl-anthracen-1-yl)isophthalonitrile (BeThPAI). Yields of the final coupling step are indicated in parentheses. Detailed reaction procedures are provided in Supplementary Note 5.

To develop a rationalized synthetic approach towards these molecules, we first convert commercially available 1,8-dichloroanthraquinone (**1)** to 1,8-dibromo-anthraquinone (**2**) to increase reactivity during subsequent coupling reactions^36^. **2** is selectively reduced to 1,8-dibromo-10-anthrone (**3**) before introduction of the phenyl ring to the anthracene core, giving 1,8-dibromo-10-phenylanthracene (**4**)^37,38^. As **4** is symmetrical, it is not possible to address the two halide groups individually during Suzuki coupling of the acceptor or donor moieties^39^. We first introduce the donor moiety to give compounds (**5-8**), and then the acceptor moiety to give the target hot exciplex molecules (see Fig. 2 and Supplementary Note 5).

### Photophysical characterization

**Fig. 3:**
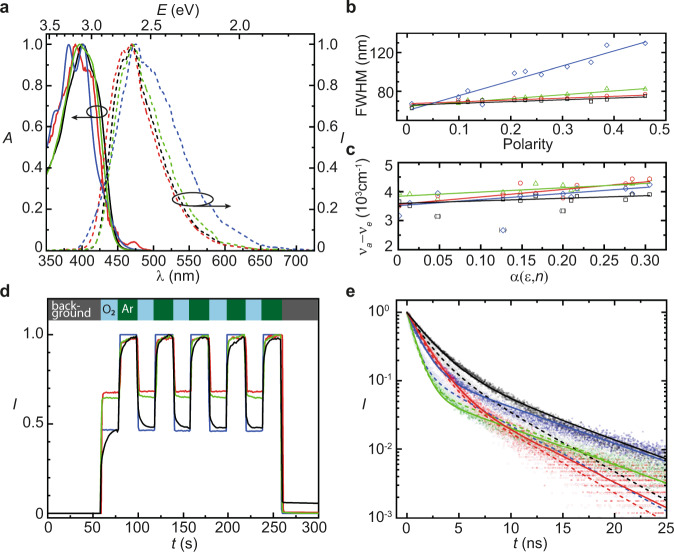
Photophysical characterization. **a** Absorption (solid line) and emission (dashed line) spectra of MeFuPAI (blue), BeFuPAI (black), MeThPAI (green), and BeThPAI (red) in chloroform solution. Spectra in different solvents are provided in Supplementary Note 1. Black arrows indicate the corresponding ordinate. **b** The fluorescence color purity, characterized by the FWHM of the emission spectrum, revealing linear dependence with the relative solvent polarity. All emitters but MeFuPAI display high color purity in polar and non-polar solvents, indicating strong LE character of the S_1_ state. **c** Lippert–Mataga plots show linear correlation between the Stokes shift (*ν*_a_−*ν*_e_) and the orientational polarizability *α*(*ε*,*n*) of the solvent; data points in parentheses are treated as outliers. **d** Normalized emission intensity under ambient (O_2_) and argon (Ar) atmosphere. **e** Time correlated single photon counting measurements of solid films under argon (solid circles, solid line fit) and oxygen (open circles, dashed line fit) to determine the photoluminescence lifetimes. Please refer to the Supplementary Fig. 2 for individual plots of each compound.

We perform absorption and photoluminescence experiments to characterize our synthesized molecules. All four molecules exhibit absorption in the near UV to violet spectrum and emission in the blue to green spectrum, which is typical for emission from an anthracene moiety (see Fig. 3a). To corroborate our claim that the S_1_ is an LE state located on the anthracene moiety, we investigate solvatochromism of our four molecules. Strong solvatochromism is expected for emission from a CT or HLCT state, while the solvent should only have a small effect on the emission from an LE state. We record absorption and photoluminescence spectra of our emitters in various solvents with different polarity. We observe that BeFuPAI, MeThPAI, and BeThPAI show narrow emission bands in polar as well as non-polar solvents with the smallest full-width at half-maximum (FWHM) in *n*-hexane (see Fig. 3b and Supplementary Fig. 1). Both, the small solvatochromic effect and the high color purity, substantiate that emission occurs from an LE state, which is beneficial for the photoluminescence quantum yield (*η*_PL_) with values between 26 and 55% (see Table 1). Interestingly, the emission spectrum of MeFuPAI reveals higher dependence on the relative polarity of the solvent than the other three molecules (see Fig. 3b)^40^. In non-polar solvents MeFuPAI demonstrates satisfying color purity and acceptable *η*_PL_ of 41% (see Fig. 3c and Table 1). To further exclude HLCT contributions in our compounds, we plot the Stokes shift (*ν*_a_−*ν*_e_) in each solvent against its orientational polarizability *α*(*ε*,*n*) to obtain Lippert–Mataga plots. Our compounds display only one component with constant slope for increasing solvent polarity (from non-polar *n*-hexane to polar acetonitrile, in Fig. 3c). By contrast, two linear components of different slope would be expected for HCLT states in this polarity range^41^. Furthermore, Lippert–Mataga plots allow estimation of the change in dipole moment upon excitation ∆*µ* = (*µ*_g_ – *µ*_e_) with *µ*_g_ and *µ*_e_ as ground and excited state dipole moments, in accordance with the Lippert–Mataga equation:

1 $${hc}\left({v}_{{{{{{\rm{a}}}}}}}-{v}_{{{{{{\rm{e}}}}}}}\right)={hc}\left({v}_{{{{{{\rm{a}}}}}}}^{0}-{v}_{\mathrm e}^{0}\right)-\frac{2{\left({\mu }_{{{\mbox{g}}}}-{\mu }_{{{\mbox{e}}}}\right)}^{2}}{{r}^{3}}\alpha \left(\varepsilon ,n\right)$$

with *r* being the Onsager radius of the compound, as determined by DFT simulations (see Supplementary Note 1), and *ν*_a_ and *ν*_e_ being the frequencies of absorption and emission. The slopes of the Lippert–Mataga plots remain below ∆*µ* < 10D and are therefore much smaller than in typical anthracene-based HLCT emitters (see Table 1)^22^. The small ∆*µ* in combination with the narrow emission bands and the negligible solvatochromic effects substantiate our claim that emission occurs from LE states in our hot exciplex molecules. To investigate participation of triplet states in the emission process, we perform oxygen-quenching experiments. In all four compounds, the blue emission is reduced when excited in ambient atmosphere, whereas when under an argon blanket the emission is strong. This effect is fully reversible (see Fig. 3d). This behavior presents a first indication that the emission occurs with participation of triplet states, as these are quenched by triplet oxygen. From the ratio of decreased intensity in the presence of oxygen versus the full intensity under argon, we estimate that triplet participation in furan-containing compounds is stronger than in thiophene-containing compounds (see Fig. 3d). Upper-level RISC in hot exciton TADF materials has been reported to be fast and practically indistinguishable from fluorescence^16^. We expect the same behavior in our hot exciplexes and investigate the photoluminescence decay of our molecules in the solid state. Indeed, the transient photoluminescence profile is composed of two fast decaying components with *τ*_pr_ = 0.80–2.0 ns, indicating prompt fluorescence, and *τ*_del_ = 5.9–8.2 ns, representing delayed fluorescence (see Fig. 3e and Table 1). The ratios of prompt to delayed fluorescence components indicate enhanced triplet contribution in furan-containing MeFuPAI and BeFuPAI over the thiophene-containing MeThPAI and BeThPAI (see Fig. 3e and Table 1). Upon exposure to oxygen, the lifetimes of the furan-containing compounds decrease significantly, whereas in the thiophene-based compounds the decay rates are almost identical under oxygen and argon (cf. dashed and solid lines in Fig. 3e). This behavior is in line with the fluorescence quenching results, where we observed less quenching in the thiophene-based compounds (cf. Fig. 3d, e). Altogether, the differences in photoluminescence lifetime under oxygen and argon are smaller than expected. This behavior can be explained by incomplete quenching by oxygen during the overall short photoluminescence lifetimes in the (sub)nanosecond regime. Photoluminescence is so abrupt that only little quenching can occur^42^. Our molecules do not exhibit the typical decrease in *η*_PL_ when cooling the material. While the energy gap for vibronic up-funneling from T_2_ to T_3_ is small, there is also a pathway for non-radiative down-funneling from T_2_ to T_1_, which becomes less probable at low temperatures. This mechanism is in line with other hot exciton materials and evokes an overall reduction in *η*_PL_ despite the TADF mechanism being still active (see Supplementary Fig. 2 and Supplementary Note 1)^43,44^.

**Table 1:** Empirical photoluminescence data and theoretical investigation of excited state energies.

	∆*µ* [D]	*η*_PL_ in CHCl_3_ [%]	*η*_PL_ solid [%]	*τ*_pr_ (Ar/O_2_) [ns]	*τ*_del_ (Ar/O_2_) [ns]	*λ*_a_^a^ [eV]/[nm]	*λ*_e_^a^ [eV]/[nm]	*E*_vert_ (S_1_)^b^ [ev]/[nm]	*E*_adiab_ (S_1_)^c^ [ev]/[nm]	*f* (S_1_)^c^
MeFuPAI	7.1	41	6	1.2/0.7	8.2/5.2	3.08/402	2.61/475	3.05/407	2.76/449	0.34
BeFuPAI	4.9	55	20	2.0/1.6	7.8/5.1	3.09/401	2.64/470	3.08/403	2.79/444	0.42
MeThPAI	6.1	8	6	0.8/0.7	8.1/7.5	3.12/397	2.63/471	3.22/385	2.88/431	0.33
BeThPAI	8.1	26	17	1.5/1.4	5.9/5.1	3.17/391	2.64/469	3.24/383	2.90/428	0.38

^a^Maxima in absorption/emission spectra measured chloroform solution. ^b^Vertical excitation energy obtained from DFT/MRCI simulations for the optimized ground state geometry. ^c^Adiabatic excitation energy computed vertically at the Tamm-Dancoff-approximated TD-PBEh-3c level.

### Characterization and evaluation of the excited states

Our empirical experiments indicate that our design principles appear correct. The emission in our molecules occurs fast, involves triplet states, and decays from an LE singlet state. However, we have no direct insight into the electronic states involved in this process. Often, time-dependent (TD)-DFT simulations are carried out to delineate the electronic properties of TADF molecules^45–47^. However, linear response TD-DFT calculations tend to underestimate triplet energies, which obscures a clear representation of our hot exciplex mechanism involving several triplet states^48^. Even individually tuned long-range corrections appear unsuitable for our hot exciplex systems, since the optimum long-range correction may vary for the individual donor, acceptor, and bridge moieties^49^. To compensate for these shortcomings, we perform density functional theory multi-reference configuration interaction (DFT/MRCI) calculations of electronic states^50,51^. The respective ground- and excited state geometries are obtained with Kohn-Sham DFT and Tamm-Dancoff-approximated TD-DFT. To evaluate the accuracy of this modeling approach we first reproduce the optical properties determined empirically. We start by performing a conformational analysis at the semiempirical GFN2-xTB level^52,53^. The obtained conformers are reoptimized and energetically reranked with the Kohn-Sham DFT-based composite method PBEh-3c. Only the lowest energy conformers are considered for our subsequent analysis (see Supplementary Fig. 3)^54^.

**Fig. 4:**
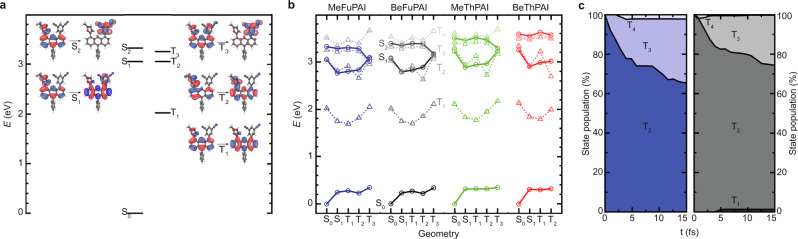
Simulation results. **a** Energy diagram of MeFuPAI at the S_0_ geometry and corresponding NTOs depicting hole and electron, allowing a differentiation into LE and CT states. **b** Visualization of singlet (circles) and triplet (triangles) energy levels (in order of decreasing contrast S_0_, S_1_, S_2_, T_1_, T_2_, T_3_, and T_4_) for all four fluorophores at the geometries of their respective S_0_, S_1_, T_1_, T_2_, and T_3_ states. Except for BeThPAI, where T_3_ is a LE state and the CT state could not be optimized. The connective line represents a guide to the eye. **c** FOMO-CASCI dynamics of the state population distribution between T_1_, T_2_, T_3_, and T_4_, upon “electrical” excitation into the T_2_ state for MeFuPAI (left) and BeFuPAI (right).

To obtain insight into the excited state characteristics, we perform natural transition orbital (NTO) analysis at the S_0_ geometry^55,56^. From this, we identify that for all molecules considered, the S_1_ state corresponds to an LE state, predominantly localized on the anthracene unit, with high *f* > 0.37 (see Fig. 4a and Supplementary Fig. 3). This is in line with our design principle and the empirical observations, and these values for *f* correspond well with the measured medium to high *η*_PL_ (see Table 1). Furthermore, the S_2_ states show strong CT character, indicating that our design concept of bringing CT and LE close together is reflected by the NTOs (see Fig. 4a and Supplementary Fig. 3). Among the triplet states, we identify T_1_ as an LE state, which is similar in character to S_1_, while T_2_ is also an LE state that is mostly located on the furan or thiophene moieties as well as on the anthracene bridge. For MeFuPAI, BeFuPAI, and MeThPAI, the T_3_ state represents a CT state, with similarity to the S_2_ state, but with a slightly more delocalized occupied NTO. For BeThPAI, this CT state is found as T_4_, while T_3_ is an LE state (see Supplementary Fig. 3). This reversal of T_3_ and T_4_ states in BeThPAI indicates that our molecules exhibit multiple high-energy triplet states of almost degenerate character, which would be in good agreement with our model of hot exciplexes.

In the next step, we refine the energies using the DFT/MRCI method (R2018 “tight” model^57^) with the TZVP basis set, based on the obtained Tamm-Dancoff-approximated TD-PBEh-3c geometries^50,51,58^. The vertical excitation energies *E*_vert_, required for reaching S_1_ from the ground state during absorption, are in good agreement with the empirical absorption *λ*_a_ obtained by UV/Vis spectroscopy (see Table 1). In much the same way, the calculated adiabatic excitation energies *E*_adia_ correspond well with the blue emission *λ*_e_ recorded by fluorescence spectroscopy (see Table 1 and Fig. 3a).

Upon excitation and (R)ISC, molecules undergo geometry adaptions due to change of the potential energy surface. As a consequence, energy levels and gaps strongly depend on the nature of the exited state geometry assumed by the respective molecule. To account for this dependence, we determine all energies of the electronic states involved in the hot exciplex mechanism in their respective minimum geometries (see Fig. 4b). At all geometries we observe the apparent large energy gaps of ~1 eV between T_1_ and T_2_ supporting our concept of hot exciplex states. In the T_3_ geometry, the energy differences between S_1_, S_2_, T_2_, and T_3_ become diminishingly small, so that these states can be considered thermally degenerate, and strong RISC is expected (see Fig. 4b). As a result of this degeneracy, we find that T_3_ in BeThPAI is an LE state and that we are unable to optimize a CT triplet state. Therefore we do not display the energy states of this geometry in Fig. 4b. To ensure that the excited states of our molecules are well represented by the ground state NTOs, we also investigate NTOs at the respective minimum geometries of relevant excited states. While we observe exchange between the T_2_ and T_3_ states in the T_3_ geometry, there is no variation in the CT or LE nature of the states (see Supplementary Figs. 3–7).

To investigate whether our model is in accordance with the observed delayed fluorescence with lifetimes of the order of only a few nanoseconds, we calculate spin–orbit coupling (SOC) matrix elements for T_2_ → S_1_ and T_3_ → S_1_ and estimate the rate of RISC *k*_RISC_ in the Condon approximation using a time-dependent approach^59^. While T_3_ → S_2_ also has a small energy gap, both states are CT states and therefore SOC is forbidden by the El-Sayed rule^60,61^. By contrast, SOC of T_2_ → S_1_ is strong, ranging between 0.35 and 2.33 cm^−1^; however, RISC is slow with *k*_RISC_ < 10_6_ s^−^^1^ (see Table 2). Apparently, in the T_2_ geometry RISC from T_2_ to S_1_ is hindered due to negative adiabatic energy gap between the two states—the T_2_ minimum is below the S_1_ minimum. While SOC for T_3_ → S_1_ is of the same order of magnitude as for T_2_ → S_1_, a positive adiabatic energy gap is present here and RISC occurs from T_3_ to S_1_ within nanoseconds (see Table 2, Fig. 4b, and Supplementary Tables 4–8).

**Table 2:** Overview of excited state energies, spin–orbit coupling, and transfer rates obtained from theoretical investigation.

	∆*E*_adia_ (T_2_–S_1_) [eV]	SOC (T_2_ → S_1_) [cm^−1^]	*k*_RISC_ (T_2_ → S_1_) [s^−1^]	∆*E*_adia_ (T_3_–S_1_) [eV]	SOC (T_3_ → S_1_) [cm^−1^]	*k*_RISC_ (T_3_ → S_1_) [s^−^^1^]	∆*E*_adia_ (T_3_–T_2_) [eV]	∆*E*_adia_ (T_2_–T_1_) [eV]
MeFuPAI	–0.10	0.35	1.1 × 10^5^	0.19	1.00	6.3 × 10^7^	0.29	0.97
BeFuPAI	–0.16	0.38	1.1 × 10^4^	0.28	0.38	1.2 × 10^7^	0.44	0.94
MeThPAI	–0.16	2.33	3.8 × 10^5^	0.28	0.93	3.1 × 10^7^	0.44	0.95
BeThPAI	–0.21	1.18	4.2 × 10^4^	–^a^	–^a^	–^a^	–^a^	0.90

^a^T_3_ in BeThPAI is a LE state. The CT states could not be optimized.

To complete the picture, we investigate what happens, once the systems are in the lowest lying hot triplet state T_2_. Excitons in the T_2_ can either down-funnel to the T_1_ state in accordance with Kasha’s rule, or up-funnel to the T_3_ state. In typical TADF compounds, vibronic coupling between T_2_ and T_3_ is on the sub-nanosecond scale and thus, significantly faster than RISC^35^. We perform ab initio multiple spawning (AIMS) simulations^62^ to obtain insight into the population dynamics in the triplet manifolds of all emitters, except BeThPAI. We start from the T_2_ state using the ab initio floating occupation molecular orbital-complete active space configuration interaction (FOMO-CASCI) approach^63^. We follow-up two different scenarios, one representing optical excitation and the other representing conditions upon electrical excitation (details are given in Supplementary Note 2).

Upon optical excitation MeFuPAI and BeFuPAI remain in the T_2_ state over the course of 90 fs, with only little exchange into T_1_ and T_3_. By contrast, in MeThPAI we observe that ~75% of the T_2_ population relaxes into the T_1_ state within 20 fs (see Supplementary Fig. 9). As a consequence, only minimal RISC is expected for MeThPAI. This result is in qualitative accordance with our conclusions drawn from the spectroscopic experiments, where MeThPAI exhibited low *η*_PL_ in solution and solid state, only minor influence of oxygen quenching, and strong and increasing non-radiative triplet decay with increasing temperature (see Fig. 3d, e, Supplementary Fig. 2 and Supplementary Table 2). By contrast, MeFuPAI and BeFuPAI displayed susceptibility to oxygen quenching, which correlates with the longer T_2_ lifetime. Appreciating these longer lifetimes in MeFuPAI and BeFuPAI and assuming that a nuclear wavepacket can partially equilibrate on the T_2_ potential energy surface, we turn to determining the minimum energy conical intersections (MECI) to estimate the long-time behavior of the remaining population on T_2_ (see Supplementary Table 9). For MeFuPAI, we find the T_1_/T_2_ MECI to be 11.5 kcal/mol above the T_2_ minimum, while it is only 7.3 kcal/mol for the T_2_/T_3_ MECI. For BeFuPAI the T_1_/T_2_ and T_2_/T_3_ MECIs are found at 9.5 and 9.6 kcal/mol, respectively. From these results we expect that the population of T_2_ can remain for periods of the order of microseconds with a greater amount of up-funneling to T_3_ in MeFuPAI and a 1:1 ratio of up- (T_3_) and non-radiative down-funneled (T_1_) states in BeFuPAI.

Applying conditions representing electrical excitation, we observe rapid up-conversion for MeFuPAI and BeFuPAI from T_2_ to T_3_. Within ~15 fs the population of T_3_ reaches about 32% for MeFuPAI and 25% for BeFuPAI (see Fig. 4c). As a consequence, vibronic up-conversion within the triplet manifold is possible in furan-containing emitters, particularly since only negligible non-radiative down-funneling to T_1_ is observed within the interval of ~15 fs.

Taken together, the empirical and theoretical experiments suggest that efficient RISC in the furan-based systems is occurring via the hypothesized T_2_ (LE) → T_3_ (CT) → S_1_ (LE) mechanism. Assuming that all up-funneled triplet excitons follow the described RISC path, we expect a radiative exciton production efficiency of approximately 44–49% in electroluminescent devices. This value is estimated by combining 1/4 of inherently radiative singlet excitons (given by spin-statistics) with the above determined 25–32% of the residual 3/4 triplet excitons that are converting fast via hot exciplex TADF.

### Electroluminescent properties

**Fig. 5:**
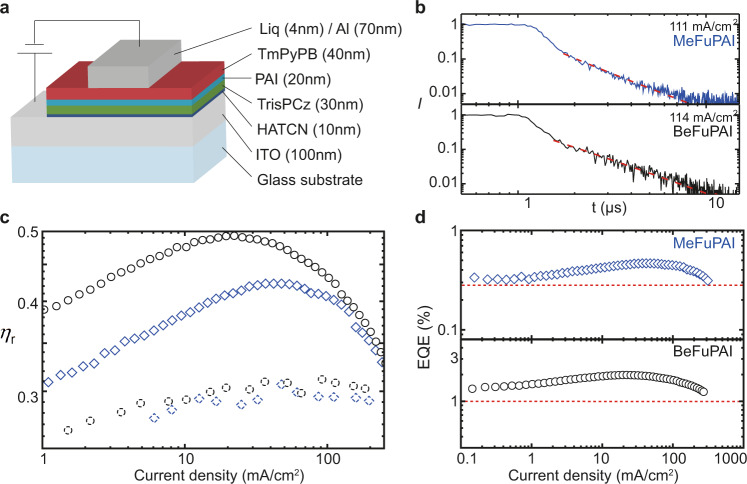
Electroluminescence characterization. **a** Schematic device structure of the OLEDs. **b** Transient electroluminescence kymographs revealing the contribution of triplet–triplet annihilation up-conversion (TTA-UC). The black line represents the data; the dashed red line is a fitting curve according to the TTA-UC model. **c** The estimated radiative production efficiency as a function of the current density showing that *η*_r_ estimated by transient electroluminescence (dotted data points) is lower than *η*_r_ determined by EQE measurements (solid data points) within the examined interval of current densities. Blue data represent MeFuPAI, black data BeFuPAI. **d** External quantum efficiencies as a function of current density. The dotted, red lines represent the respective maximal EQEs for conventional fluorescent OLEDs harvesting only singlet excitons.

To verify that the hot exciplex mechanism is also present in its intended application of light-emitting diodes, we fabricate devices and determine their electroluminescence characteristics. In this context, we focus on oxygen-containing emitters MeFuPAI and BeFuPAI as they exhibit superior *η*_PL_, distinct SOC, and better T_2_ to T_3_ up-funneling compared to MeThPAI and BeThPAI. We fabricate OLEDs with neat emitter layers (see Fig. 5a). The electroluminescence spectra are almost identical to the photoluminescence spectra and independent of the current density between 1 and 100 mA/cm^2^ (see Supplementary Fig. 10). We obtain EQEs of 0.5% for MeFuPAI and 2% for BeFuPAI. The radiative exciton production efficiencies *η*_r_ of the MeFuPAI and BeFuPAI devices are determined as 45% and 50%, respectively, using

2 $${{\mbox{EQE}}}=\gamma \cdot {\eta }_{{{\mbox{r}}}}\cdot {\eta }_{{{\mbox{PL}}}}\cdot {\eta }_{{{\mbox{O}}}}$$

with *γ* as the carrier balance factor being close to unity, *η*_PL_ in the solid state (6% and 20%, respectively; see Table 1), and *η*_O_ as the outcoupling efficiency of around 20%. As expected, *η*_r_ exceeds the 25% limit in radiative exciton production efficiencies of conventional fluorescent OLEDs, and coincides with the interval estimated by FOMO-CASCI simulations.

We perform transient electroluminescence to verify the hot exciplex mechanism in electroluminescent devices and study the potential contribution of triplet–triplet interactions, which are to be expected in neat emitter layers. The transient emission spectra for these devices exhibit a prompt component, accounting for ~80% of the emission in the nanosecond regime, and a delayed component of ~20% in the microsecond regime (see Fig. 5b and Supplementary Fig. 11). This delayed component is surprising at first, acknowledging that the hot exciplex based TADF is expected to occur on the nanosecond regime. However, the delayed component fits a recently described model for triplet–triplet annihilation up-conversion (TTA-UC), and can therefore not be assigned to the hot exciplex emission (see red dashed lines for TTA-UC fit in Fig. 5b)^22,64^:

3 $$\frac{{I}_{{{\mbox{del}}}}}{{I}_{{{\mbox{pr}}}}}=4{\eta }_{{{\mbox{r}}}}-1.$$

The contribution of TTA-UC is plausible because the emitter concentration is high and intermolecular emitter–emitter interactions are therefore allowed. As a result, our hot exciplex RISC pathway is so fast, that it needs to be assigned to the prompt emission decay. From the difference between *η*_r, EQE_ = 45–50% determined from EQE (Eq. 2) and *η*_r,transEL_ = 30% obtained from transient electroluminescence (Eq. 3) (see Fig. 5c), we can derive the contribution of hot exciplexes to the overall emission *I*_HE_ by

4 $${I}_{{{\mbox{HE}}}}=\frac{1}{4{\eta }_{{{\mbox{r, transEL}}}}}-\frac{1}{4{\eta }_{{{\mbox{r, EQE}}}}}$$

(see Supplementary Note 4 for derivation of Eq. 4).

This analysis shows that hot exciplexes account for up to 24% and 32% of the electroluminescence in MeFuPAI and BeFuPAI devices, respectively. The presence of a hot exciplex RISC mechanism is further substantiated by the diminishing contribution of TTA-UC for decreasing current densities (Fig. 5c). Even at low current density the observed EQEs exceed the fluorescent OLED limit (see Fig. 5d). As described above, a hot exciton mechanism utilizing HLCT states would show roll-off at high current density. By contrast, our hot exciplex pathway contributes to the electroluminescence process across the entire range of current densities (see Fig. 5d).

In summary, we have devised a design strategy that produces a so far unexplored hot exciplex mechanism yielding an LE state as the emissive singlet. By capitalizing on a molecular toolbox design, the respective donor and acceptor moieties can be varied, in the future, to evoke emitters with superior *η*_PL_ and EQE. Combined with the inherently fast RISC process, short luminescence lifetimes, as well as high color purity and low efficiency roll-off, hot exciplexes are primed for advanced optoelectronic applications such as blue-emitting OLEDs and might contribute to new material concepts for organic laser diodes.

## Methods

Detailed description of the used methods, synthetic procedures, and details on the computational study are provided within Supplementary Notes 2, and 5, available in the online version of the paper.

## Supplementary information

Supplementary Information

## Data Availability

The data generated in this study are provided as Source Data file. Source data are provided with this paper.

## Source data

Source Data

## Code availability

All software used in this study is third–party software and obtained online: CREST code is available at https://github.com/grimme-lab/crest, xTB Version 6.4.0 is available at https://github.com/grimme-lab/xtb; the Turbomole 7.4.1 software package is available at https://www.turbomole.org/; the DFT/MRCI program is available at https://www.theochem.hhu.de/en/translate-to-english-forschung/translate-to-english-dft/mrci; VIBES code is available upon request from C. Marian (https://www.theochem.hhu.de/en/translate-to-english-forschung); FMS90 code (AIMS) is available upon request from Todd J. Martinez (https://mtzweb.stanford.edu/contact); TeraChem is available at http://www.petachem.com/products.html.
