# Supplementary material for: Hot exciplexes in U-shaped TADF molecules with emission from locally excited states
Source: Nat Commun. 2021 Oct 26;12:6179. doi: 10.1038/s41467-021-26439-w (PMC8548444; doi:10.1038/s41467-021-26439-w)
Supplement: Supplementary file 1 — Supplementary Information [file 41467_2021_26439_MOESM1_ESM.pdf]

Hot exciplexes in U-shaped TADF molecules with emission from locally excited states

Supplementary Information

A. Lennart Schleper, Kenichi Goushi, Christoph Bannwarth, Bastian Haehnle, Philipp J. Welscher, Chihaya Adachi, and Alexander J. C. Kuehne

Supplementary Note 1: Optical spectroscopy 1
Supplementary Note 2: Computational study of the hot exciplex emitters 5
Supplementary Note 3: Electroluminescence 14
Supplementary Note 4: Calculation of I_{HE} 14
Supplementary Note 5: Experimentals 15
Supplementary Note 6: NMR spectra 19
Supplementary References 23

Supplementary Note 1: Optical spectroscopy

Supplementary Table 1 | Solvents used for the Lippert-Mataga plots.

Solvent	Polarity	Orientational polarizability
Acetone	0.355	0.284
Acetonitrile	0.460	0.305
Chloroform	0.259	0.148
Dichloromethane	0.309	0.217
Diethylamine	0.145	0.127
Diethyl ether	0.117	0.167
Dimethylformamide	0.386	0.276
Ethyl acetate	0.228	0.200
n-Hexane	0.009	0.0012
Tetrahydrofuran	0.207	0.210
Toluene	0.099	0.014
Triethylamine	0.043	0.048

The Onsager radius *r* – required for evaluation of the Lippert-Mataga plots – is estimated from DFT simulations on the B3LYP/6-31+G(d) level using the *Volume* keyword in the Gaussian09 software program.[1]

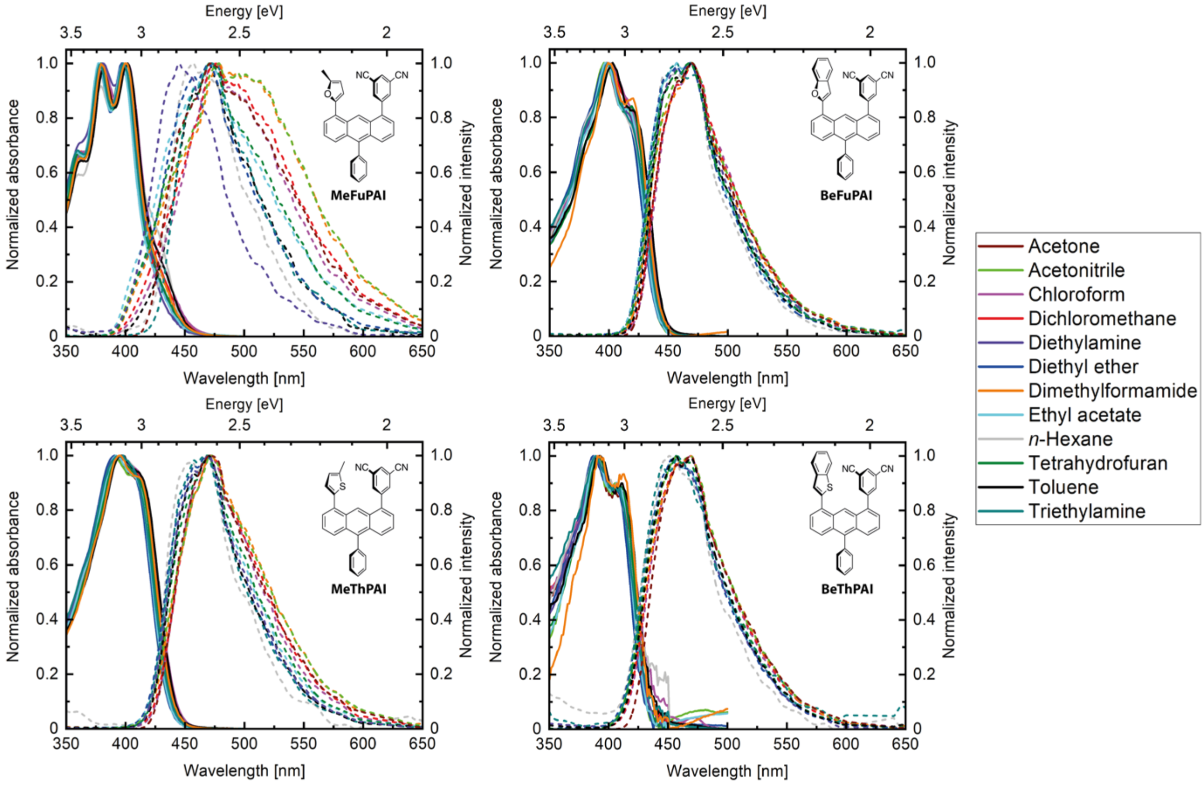

Supplementary Figure 1 | Optical spectroscopy in different solvents. Absorption (solid line) and photoluminescence (dashed line) spectra of MeFuPAI, BeFuPAI, MeThPAI, and BeThPAI in different solvents as indicated by the color code.

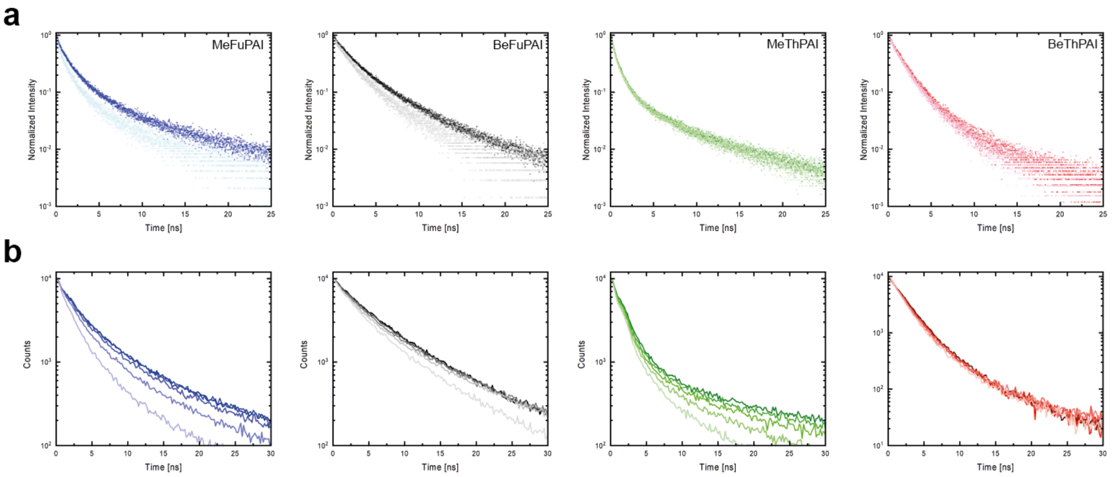

Supplementary Figure 2 | Transient photoluminescence spectra. a, TCSPC measurements of MeFuPAI, BeFuPAI, MeThPAI, and BeThPAI solid films under argon (solid data points) and oxygen (brighter, empty data points) atmosphere. **b**, Lifetime measurements under argon at 100 K, 150 K, 200 K, 250 K, and 300 K (from dark to bright).

We derive rate constants for the relevant decay processes of the excited state from our temperature dependent transient photoluminescence experiments. We fit the data with a bi-exponential decay $y(x) = A_{\mathrm{PF}}\, e^{-\frac{x}{\tau_{\mathrm{PF}}}} + A_{\mathrm{DF}}\, e^{-\frac{x}{\tau_{\mathrm{DF}}}}$ to consider the prompt and delayed components. From these fits we can calculate the quantum efficiencies of prompt η_{PF} and delayed fluorescence η_{DF} for each temperature *via*:[2]

$$\eta_{\mathrm{PF}} = \frac{(A_{\mathrm{PF}} + A_{\mathrm{DF}})\, k_{\mathrm{DF}}}{A_{\mathrm{PF}}\, k_{\mathrm{DF}} + A_{\mathrm{DF}}\, k_{\mathrm{PF}}}\, \eta_{\mathrm{PL}}$$

$$\eta_{\mathrm{DF}} = \frac{(k_{\mathrm{PF}} - k_{\mathrm{DF}})\, A_{\mathrm{DF}}}{A_{\mathrm{PF}}\, k_{\mathrm{DF}} + A_{\mathrm{DF}}\, k_{\mathrm{PF}}}\, \eta_{\mathrm{PL}}$$

$$\text{with } k_{\mathrm{DF}} = \frac{1}{\tau_{\mathrm{DF}}} \text{ and } k_{\mathrm{PF}} = \frac{1}{\tau_{\mathrm{PF}}}.$$

From the individual components to the overall photoluminescence quantum yield ($\eta_{\mathrm{PL}} = \eta_{\mathrm{PF}} + \eta_{\mathrm{DF}}$) we can now determine the radiative decay rate of the singlet state ($S_1 \rightarrow S_0$):

$$k_{\mathrm{rad}}^{\mathrm{S}} = \frac{\eta_{\mathrm{PF}}}{\tau_{\mathrm{PF}}}$$

Delayed fluorescence is a process, in which consecutive intersystem crossing (ISC) and reverse intersystem crossing (RISC) occurs and the excited states may exchange several times between S_1 and T_2 before following one of the possible decay pathways. Therefore, we cannot derive a simple equation as for prompt fluorescence. Delayed fluorescence needs to be expressed as a sum of the possible decay processes:[3,4]

$$\eta_{\mathrm{DF}} = \eta_{\mathrm{RISC}}\, \eta_{\mathrm{ISC}}\, \eta_{\mathrm{PF}} + \eta_{\mathrm{RISC}}^2\, \eta_{\mathrm{ISC}}^2\, \eta_{\mathrm{PF}} + \eta_{\mathrm{RISC}}^3\, \eta_{\mathrm{ISC}}^3\, \eta_{\mathrm{PF}} + \eta_{\mathrm{RISC}}^4\, \eta_{\mathrm{ISC}}^4\, \eta_{\mathrm{PF}} + \cdots$$

$$\eta_{\mathrm{DF}} = \eta_{\mathrm{PF}} \sum_{1}^{n} (\eta_{\mathrm{RISC}}\, \eta_{\mathrm{ISC}})^n$$

$$\eta_{\mathrm{DF}} = \eta_{\mathrm{PF}} \sum_{0}^{n} (\eta_{\mathrm{RISC}}\, \eta_{\mathrm{ISC}})^n - \eta_{\mathrm{PL}}$$

This geometric series converges for $\eta_{\mathrm{RISC}}\, \eta_{\mathrm{ISC}} < 1$ to the much simpler form:

$$\eta_{\mathrm{DF}} = \eta_{\mathrm{PF}} \frac{1}{1 - \eta_{\mathrm{RISC}} \eta_{\mathrm{ISC}}} - \eta_{\mathrm{PF}}$$

Following analogous approaches for $\eta_{\mathrm{nr}}^{\mathrm{S}}$ and $\eta_{\mathrm{nr}}^{\mathrm{T}}$, we can setup a master equation for all processes where the sum of all radiative and non-radiative decay processes is 1:

$$\sum\eta_{\mathrm{rad}} + \sum\eta_{\mathrm{nr}} = 1 = \frac{\eta_{\mathrm{PF}} + \eta_{\mathrm{nr}}^{\mathrm{S}} + \eta_{\mathrm{ISC}} \times \eta_{\mathrm{nr}}^{\mathrm{T}}}{1 - \eta_{\mathrm{ISC}} \times \eta_{\mathrm{RISC}}}$$

However, the individual non-radiative processes (non-radiative decay on the singlet ($_{\mathrm{nr}}^{\mathrm{S}}$) and triplet side ($_{\mathrm{nr}}^{\mathrm{T}}$), as well as ISC and reverse intersystem crossing RISC) remain elusive.

In contrast to established methods, where $k_{\mathrm{nr}}^{\mathrm{T}} \approx 0$ or $k_{\mathrm{rad}}^{\mathrm{S}} \gg k_{\mathrm{RISC}} \gg k_{\mathrm{nr}}^{\mathrm{T}}$ can be assumed to simplify equations, our system does not offer this luxury. Due to the presence of a low-energy T_1 state, we cannot ignore non-radiative decay within the triplet manifold in our hot exciton emitters. Furthermore, we expect RISC to be competitive with prompt fluorescence. As a consequence, established methods for the determination of RISC fail.[2]

Here we make two other assumptions that are more applicable to our hot exciplex system:

1. we assume non-radiative decay processes to become irrelevant at low temperatures so that $\eta_{\mathrm{nr}}^{\mathrm{S}} \approx 0$ for $T = 100$ K,
2. we only consider photo-excitation, which can be regarded as adiabatic so that there is excess energy in the excited state that allows us to assume ISC to be temperature independent.

Following these assumptions we can determine η_{ISC} when we set $\eta_{\mathrm{PF}} + \eta_{\mathrm{ISC}} + \eta_{\mathrm{nr}}^{\mathrm{S}} = 1$:

$$\eta_{\mathrm{ISC}} = 1 - \eta_{\mathrm{PF}} \text{ at } 100\text{ K}.$$

Following assumption 2 we can now determine η_{RISC} for all temperatures.

We determine the rates for RISC and $_{\mathrm{nr}}^{\mathrm{T}}$ as follows:[3,4]

$$k_{\mathrm{RISC}} = \frac{1}{\tau_{\mathrm{DF}}} \frac{\eta_{\mathrm{RISC}}}{(1 - \eta_{\mathrm{ISC}})\,\eta_{\mathrm{RISC}}}$$

$$k_{\mathrm{nr}}^{\mathrm{T}} = \frac{k_{\mathrm{RISC}}}{\left(\eta_{\mathrm{nr}}^{\mathrm{T}}\right)^{-1} - 1}$$

and $\eta_{\mathrm{nr}}^{\mathrm{T}}$ can be determined from the master equation. The obtained values reveal that the RISC process is indeed thermally activated, as required for TADF. The overall decreased η_{F} at elevated temperatures is caused by the increased influence of non-radiative decay (see Supplementary Table 2).

Supplementary Table 2 | Temperature-dependent kinetics. Photoluminescence quantum yield and rate constants for all relevant photoluminescence decay process at different temperatures.

	T (K)	ϕ_{PL} (%)	k_{rad} (ns^{-1})	k_{nr}^{S} (ns^{-1})	k_{ISC} (ns^{-1})	k_{RISC} (ns^{-1})	k_{nr}^{T} (ns^{-1})
MeFuPAI	100	20.3	0.044	0	0.33	0.82	0.88
	150	17.7	0.038	0.005	0.33	0.80	0.90
	200	13.5	0.031	0.014	0.33	0.86	0.96
	250	10.6	0.030	0.019	0.33	0.96	1.27
	300	7.4	0.029	0.026	0.33	1.28	2.04
BeFuPAI	100	38.1	0.062	0	0.23	0.49	0.38
	150	39.1	0.064	0	0.23	0.48	0.38
	200	34.3	0.058	0.008	0.23	0.46	0.32
	250	28.5	0.051	0.020	0.23	0.47	0.29
	300	19.9	0.042	0.031	0.23	0.58	0.42
MeThPAI	100	9.7	0.026	0	0.47	0.64	0.69
	150	9.0	0.027	0.001	0.47	0.74	0.85
	200	7.5	0.025	0.004	0.47	0.87	1.21
	250	5.9	0.023	0.007	0.47	1.30	2.31
	300	4.5	0.021	0.010	0.47	1.91	4.73
BeThPAI	100	19.3	0.070	0	0.35	0.67	3.36
	150	18.8	0.070	0.003	0.35	0.71	3.25
	200	17.9	0.067	0.007	0.35	0.66	2.77
	250	16.7	0.066	0.011	0.35	0.58	2.46
	300	15.3	0.065	0.018	0.35	0.77	2.81

Supplementary Note 2: Computational study of the hot exciplex emitters

Natural Transition Orbitals

During conformational analysis of our four emitters we find local energy minima for conformers with regard to rotation around the donor-anthracene bond. We determine two preferred conformers – one with the heteroatom of the donor pointing towards the isophthalonitrile acceptor ("in") and one with the heteroatom pointing away from the isophthalonitrile ("out"). While furan-containing compounds prefer the "out" conformation, "in" conformers are preferred from the thiophene-containing compounds. In the main manuscript we focus on the lowest-energy conformers which are MeFuPAI-"out", BeFuPAI-"out", MeThPAI-"in", and BeThPAI-"in". Since the conformers are found within an energy window of 1 kcal/mol (at the PBEh-3c level), we expect the presence of both conformers at room temperature we calculate Natural Transition Orbitals (NTOs) for both conformers. However, we observe only a small effect of the conformation on the character of the NTOs. Hence, we only used the most stable conformer for the subsequent analysis. We verified that the state ordering for the lowest three singlet and triplet states agrees with DFT/MRCI at the S_0 geometry (see below). For better comprehensibility we assign all states as LE or CT states. However, in most cases the states, as they are depicted by the NTOs, are no pure LE or CT states, but show partially overlapping holes and electrons.

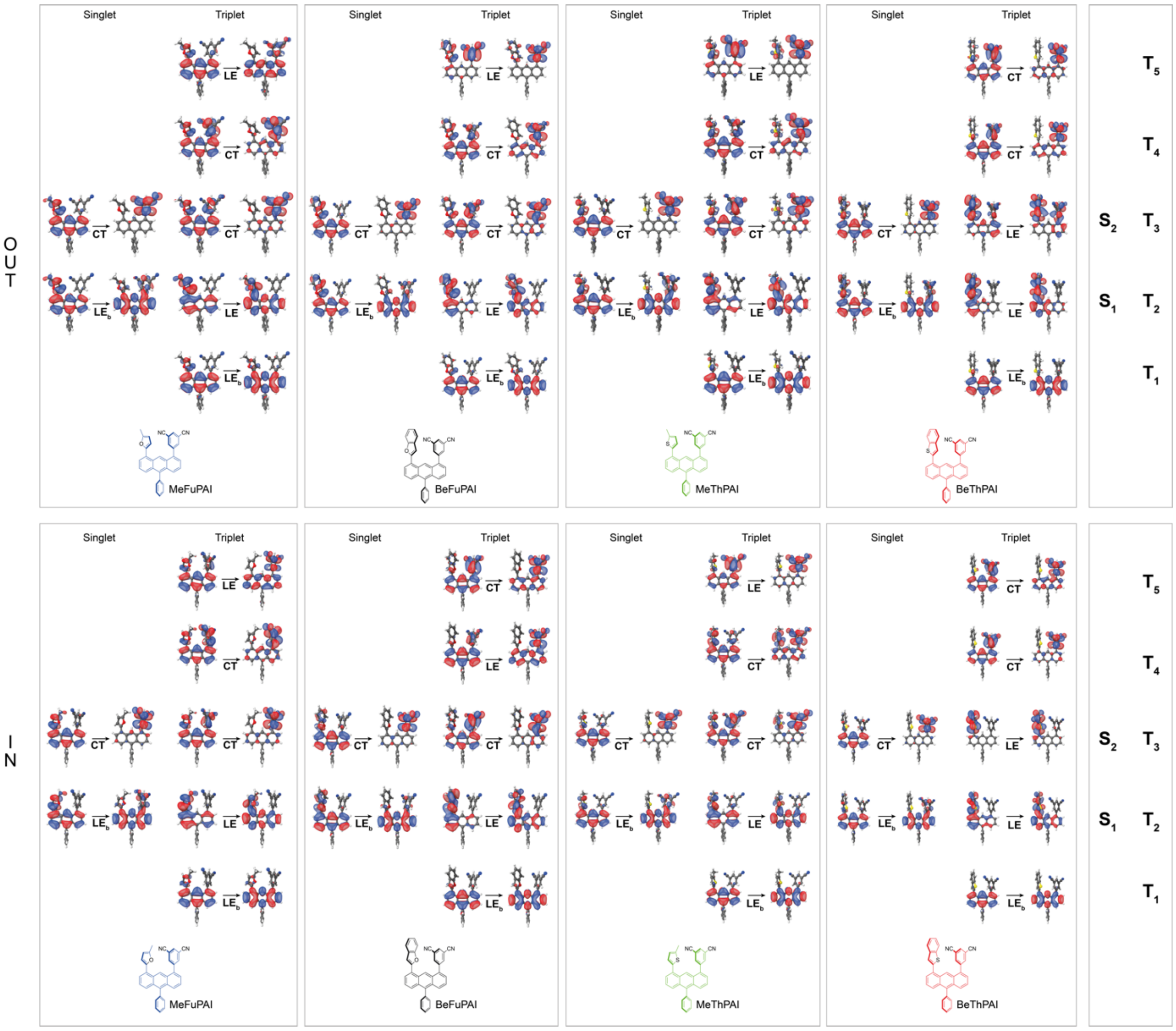

Supplementary Figure 3 | NTOs at the S_0 geometry. Natural Transition Orbitals (determined *via* Tamm-Dancoff-approximated TD-DFT calculations employing the PBEh-3c density functional approximation) of "out" and "in" conformers of all four emitters – in energetic order from S1 (left columns, bottom) to S2 (left columns, top) and T1 (right columns, bottom) to T5 (right columns, top). While the "out" conformers are lowest in energy for the methylfuranyl derivatives, the "in" conformers are found to be lower for the

thiophenyl derivatives. These conformers (“out” for furanyl, “in” for thiophenyl) are used in the main manuscript. (colors adapted to match the manuscript and labels added)

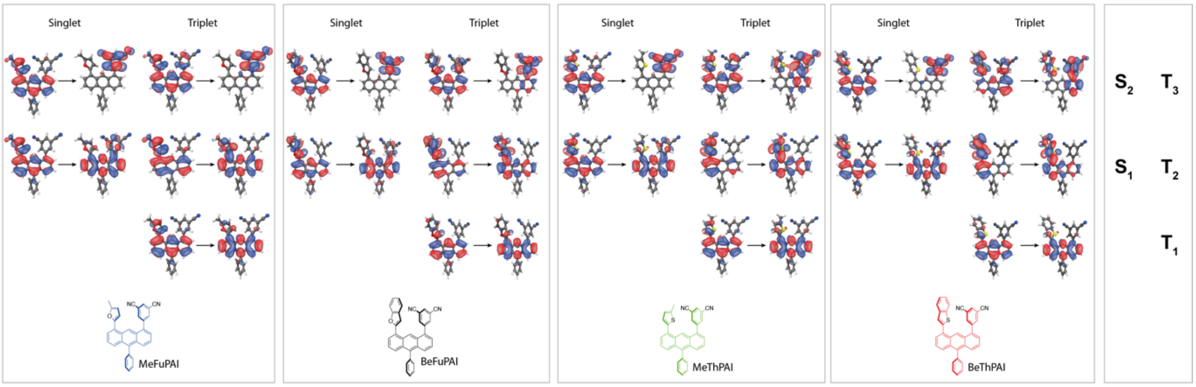

Supplementary Figure 4 | NTOs at the S_1 geometry. Natural Transition Orbitals of the lowest-energy conformers (“out” for MeFuPAI and BeFuPAI; “in” for MeThPAI and BeThPAI) at the optimized S_1 geometry – in energetic order from S1 (left columns, bottom) to S2 (left columns, top) and T1 (right columns, bottom) to T3 (right columns, top).

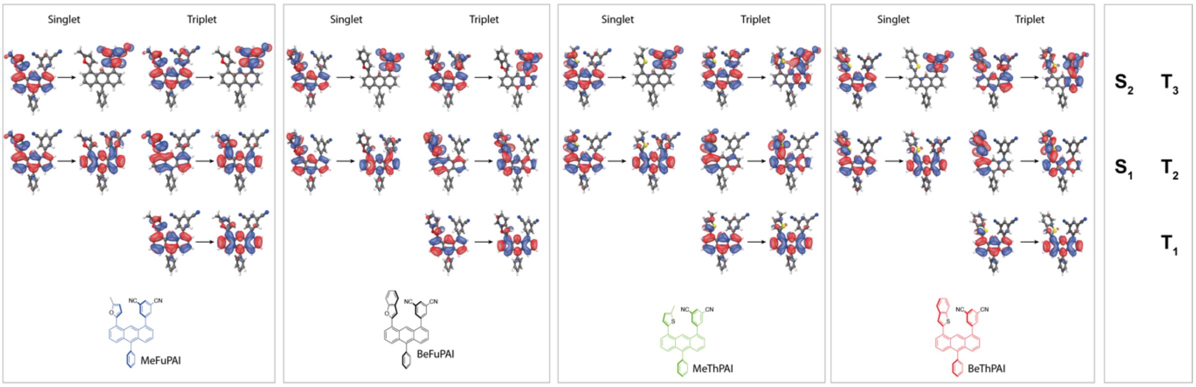

Supplementary Figure 5 | NTOs at the T_1 geometry. Natural Transition Orbitals of the lowest-energy conformers (“out” for MeFuPAI and BeFuPAI; “in” for MeThPAI and BeThPAI) at the optimized T_1 geometry – in energetic order from S1 (left columns, bottom) to S2 (left columns, top) and T1 (right columns, bottom) to T3 (right columns, top).

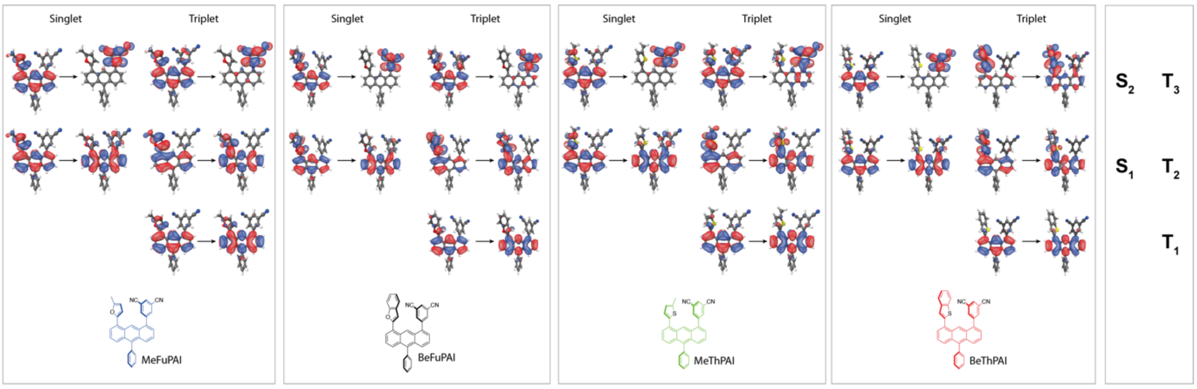

Supplementary Figure 6 | NTOs at the T_2 geometry Natural Transition Orbitals of the lowest-energy conformers (“out” for MeFuPAI and BeFuPAI; “in” for MeThPAI and BeThPAI) at the optimized T_2 geometry – in energetic order from S1 (left columns, bottom) to S2 (left columns, top) and T1 (right columns, bottom) to T3 (right columns, top).

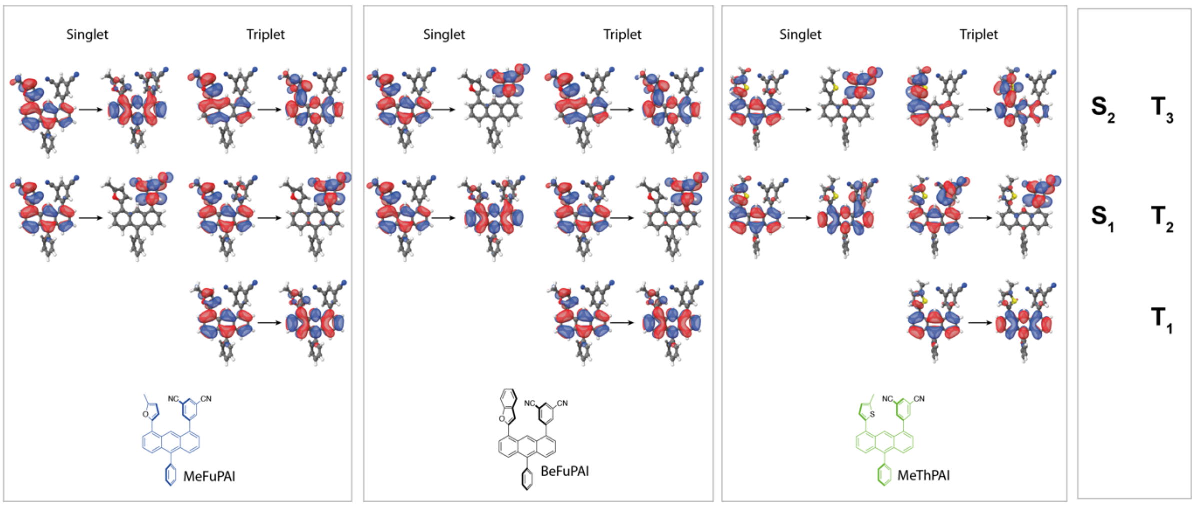

Supplementary Figure 7 | NTOs at the T_3 geometry Natural Transition Orbitals of the lowest-energy conformers ("out" for MeFuPAI and BeFuPAI; "in" for MeThPAI) at the optimized T_3 geometry – in energetic order from S1 (left columns, bottom) to S2 (left columns, top) and T1 (right columns, bottom) to T3 (right columns, top). An optimized geometry for BeThPAI could not be obtained.

It is typically assumed, that upon electric excitation of a compound an electron is withdrawn from its highest occupied molecular orbital (HOMO) and another electron is injected into its lowest unoccupied molecular orbital (LUMO). To ensure that electric excitation of S_1 and T_2 is possible – directly or after internal conversion from a higher excited state – we investigate the location of HOMO and LUMO as well as their contributions to the excited states. We observe that HOMO and LUMO both reside mainly on the anthracene bridge. While the HOMO is partially extended to the furanyl/thiophenyl donor, the LUMO shows contributions from the isophthalonitrile acceptor (see Supplementary Figure 8). As expected HOMO and LUMO contributions are dominating in the lower excited states, implying that electrical excitation of S1 and T_2 is possible (see Supplementary Table 3).

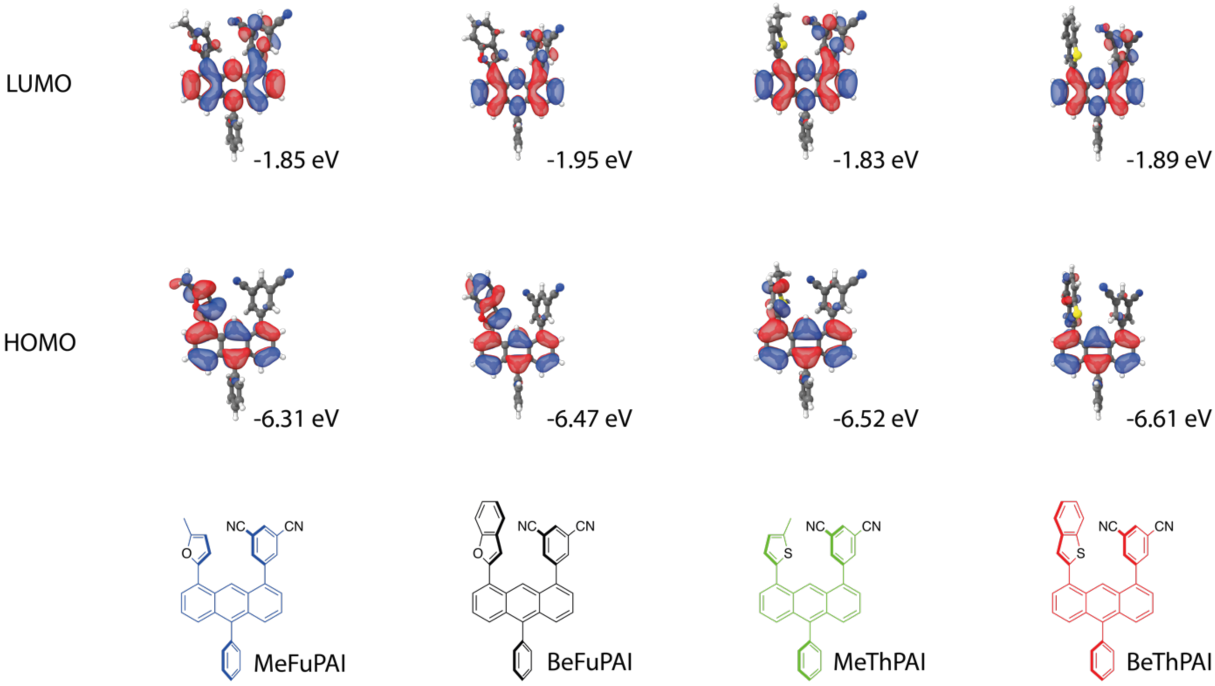

Supplementary Figure 8 | Frontier orbitals. HOMO and LUMO of all four emitters in their S_0 minimum geometry, and the respective (calculated) energies.

Supplementary Table 3 | Orbital contributions. Orbital contributions (>0.1) to the excited states at the S_0 geometry as determined *via* DFT/MRCI (for calculation details see the section *"Calculation of spin-forbidden nonradiative transitions"*).

	MeFuPAI	BeFuPAI	MeThPAI	BeThPAI
S_1	HOMO → LUMO (0.85)	HOMO → LUMO (0.85)	HOMO → LUMO (0.85)	HOMO → LUMO (0.85)
S_2	HOMO → LUMO+1 (0.83)	HOMO → LUMO+1 (0.80)	HOMO → LUMO+1 (0.78)	HOMO → LUMO+1 (0.74)
T_1	HOMO → LUMO (0.73)	HOMO → LUMO (0.73)	HOMO → LUMO (0.76)	HOMO → LUMO (0.78)
T_2	HOMO-1 → LUMO (0.41)<br>HOMO → LUMO+4 (0.21)	HOMO-1 → LUMO (0.34)<br>HOMO → LUMO+4 (0.16)<br>HOMO → LUMO+3 (0.11)	HOMO-1 → LUMO (0.34)<br>HOMO → LUMO+2 (0.18)<br>HOMO → LUMO+4 (0.13)<br>HOMO-6 → LUMO (0.11)	HOMO → LUMO+2 (0.15)<br>HOMO-1 → LUMO (0.13)<br>HOMO-5 → LUMO (0.10)
T_3	HOMO → LUMO+1 (0.79)	HOMO → LUMO+1 (0.74)	HOMO → LUMO+1 (0.71)	HOMO → LUMO+1 (0.62)

Calculation of spin-forbidden nonradiative transitions

To simulate the intersystem crossing (ISC) and reverse ISC (RISC) rates between the energetically close lying triplet and singlet states, we employ the two-state time-dependent approach of Marian and coworkers in the Condon approximation based on harmonic oscillators.[5] For this approach, the geometries of the respective electronic states need to be optimized and the harmonic frequencies to be computed at the respective minima.

Since the state ordering at the S_0 geometry is found to be in agreement with high level density functional theory-based multi-reference configuration interaction (DFT-MRCI)[6,7] calculations (except for BeThPAI, where the triplet CT state is T_3 with DFT/MRCI), we perform these geometry optimizations also at the TDA-TDDFT-PBEh-3c level. We include all states through the respective CT state of each spin manifold. To avoid ambiguity in the case of state flips during the optimizations, we denote the included states as ^{1}LE and ^{1}CT for the excited singlet and ^{3}LE1, ^{3}LE2, and ^{3}CT for the triplet states (only ^{1}LE, ^{3}LE1, and ^{3}LE2 in the case of BeThPAI) in the following. It is noted that at the TDA-TDDFT-PBEh-3c level, the ^{1}CT geometry optimization leads to a crossing of the ^{1}CT and ^{1}LE states, which complicates the geometry optimization of the ^{1}CT state. For technical reasons, we thus preoptimize the ^{1}CT state at the TDA-TDDFT-B3LYP-D3(BJ)[8–14] level first, since the ^{1}CT state is obtained as S_1 here, thus simplifying the optimization of that state. We then continued with the TDA-TDDFT-PBEh-3c optimization, which is now also possible, since the ^{1}CT has dropped to the S_1 position at the TDA-TDDFT-B3LYP-D3(BJ)-preoptimized geometry. We use the permanent dipole moment of the state to distinguish it from the LE states – the CT states show static dipole moments that are larger in magnitude by at least 10 Debye compared to the LE states. For BeThPAI, optimizations of the ^{1}CT and the ^{3}CT state geometries is not possible, due to state slips during the optimization.

We verify each geometry as a minimum by numerical Hessian calculations and use the corresponding harmonic frequencies in the time-dependent harmonic oscillator approach as implemented in the VIBES program.[15] All optimized geometries are given in a zip file with this supporting information.

Instead of using the adiabatic energy gap computed at the TDA-TDDFT directly, higher level DFT-MRCI[6,7] calculations employing the R2018[16] Hamiltonian with "tight" parametrization (for $esel=0.8 a.u.) and Ahlrichs' TZVP basis set[17] are used to compute the electronic energies at each geometry. The DFT-MRCI method is a comparably efficient but highly accurate excited state electronic structure method and often on a par with the CC2 approximate coupled cluster method.[18,19] The DFT-MRCI/TZVP//TDA-TDDFT-PBEh-3c are then used to specify the adiabatic energy gap of the two states between which the ISC rate has been calculated with the VIBES program. Furthermore, the spin-orbit coupling matrix elements between the spin-pure DFT-MRCI/TZVP states are evaluated at the respective initial state geometry. The electronic energies obtained at this level are given in Supplementary Tables 4–7. The equation for the (R)ISC rate in the Condon approximation is given as

$$k_{\mathrm{(R)ISC}}^{i\to f} = \left(< \Psi_i \middle| \hat{H}_{\mathrm{SOC}} \middle| \Psi_f >\right)^2 k_{\mathrm{vib}}^{i\to f},$$

with the vibrational component being computed via numerical integration of the time correlation function:

$$k_{\mathrm{vib}}^{i\to f} = \int_{-\infty}^{\infty} G(t) e^{it[\Delta E_{if}^{0} + E_i^{\mathrm{ZPVE}}]} \mathrm{d}t.$$

Here *G(t)* is the generation function (see Ref.[5]), ΔE_{if}^{0} is the adiabatic energy difference between the initial and final states (computed at the DFT-MRCI/TZVP//TDA-TDDFT-PBEh-3c level in this work), and the last term is the zero-point vibrational energy (ZPVE) of the initial state (computed at the TDA-TDDFT-PBEh-3c level in this work). We test the method parameters for the ^{3}LE2 to ^{1}LE RISC in MeFuPAI and find the RISC rate to be very insensitive with respect to them. We use a time interval of 100 fs, a Gaussian damping width of 1 cm^{-1} and 8192 grid points for the time integration in all cases. The RISC rates are computed for a temperature of 300 K throughout.

We note that for all systems, we find that at the TDA-TDDFT-PBEh-3c-optimized ^{3}CT geometry, the ^{3}CT state remains in the T_3 position with TDA-TDDFT-PBEh-3c, while it the drops below the ^{3}LE2 state (i.e., it takes the T_2 position) at the DFT-MRCI/TZVP//TDA-TDDFT-PBEh-3c level. We notice furthermore that at the DFT/MRCI/TZVP level, the ^{3}CT energy is always lower at the TDA-TDDFT-PBEh-3c ^{1}CT state minimum than on the respective ^{3}CT state minimum geometry. This may be due to stronger mixing with LE states in the triplet manifold at the TDA-TDDFT-PBEh-3c level of theory. Given that for pure CT states, the singlet and triplet states are expected to be isoenergetic, we also consider the ^{1}CT state geometry to be a plausible geometry to represent the ^{3}CT state minimum. For comparison, we decide to consider the TDA-TDDFT-PBEh-3c ^{1}CT and ^{3}CT minimum geometries (and their harmonic frequencies), for the DFT/MRCI single-point calculations to estimate the RISC from the ^{3}CT state (cf. Supplementary Table 8). In all cases, we identify the respective DFT-MRCI states by visual inspection of the dominantly contributing orbitals to the respective excitations.

The Turbomole program[20] is used to conduct all DFT and TDA-TDDFT calculations, while the standalone mrci program of Marian and coworkers along with related tools is used to perform the DFT-MRCI calculations (based on the BHLYP[8,9,12] orbitals generated with Turbomole) and calculations of spin-orbit coupling matrix elements.

Supplementary Table 4 | Energy landscape of MeFuPAI. Electronic state energies computed for MeFuPAI at the DFT-MRCI/TZVP level on the corresponding PBEh-3c geometries. The TDA-TDDFT formalism has been used for the excited state geometry calculations. The relative energies w.r.t. the S_0 energy at the S_0 geometry are given in electron volts (eV) on the right next to the total energy (in Hartree) of the respective state.

MeFuPAI	Minimum geometry											
Electronic state (designation in the main manuscript)	S_0		^{1}LE (S_1)		^{1}CT (S_2)		^{3}LE1 (T_1)		^{3}LE2 (T_2)		^{3}CT (T_3)	
S_0	-1453.8766	0.00	-1453.8676	0.25	-1453.8632	0.37	-1453.8664	0.28	-1453.8683	0.23	-1453.8639	0.35
^{1}LE (S_1)	-1453.7646	3.05	-1453.7753	2.76	-1453.7627	3.10	-1453.7738	2.80	-1453.7727	2.83	-1453.7632	3.09
^{1}CT (S_2)	-1453.7547	3.32	-1453.7562	3.28	-1453.7663	3.00	-1453.7552	3.30	-1453.756	3.28	-1453.766	3.01
^{3}LE1 (T_1)	-1453.8025	2.02	-1453.8123	1.75	-1453.801	2.06	-1453.8147	1.69	-1453.8098	1.82	-1453.8013	2.05
^{3}LE2 (T_2)	-1453.7647	3.05	-1453.7734	2.81	-1453.7633	3.08	-1453.7695	2.91	-1453.7788	2.66	-1453.7636	3.08
^{3}CT (T_3)	-1453.7571	3.25	-1453.7585	3.21	-1453.7686	2.94	-1453.7574	3.25	-1453.7584	3.22	-1453.7684	2.95

Supplementary Table 5 | Energy landscape of BeFuPAI. Electronic state energies computed for BeFuPAI at the DFT-MRCI/TZVP level on the corresponding PBEh-3c geometries. The TDA-TDDFT formalism has been used for the excited state geometry calculations. The relative energies w.r.t. the S_0 energy at the S_0 geometry are given in electron volts (eV) on the right next to the total energy (in Hartree) of the respective state.

BeFuPAI	Minimum geometry											
Electronic state (designation in the main manuscript)	S_0		^{1}LE (S_1)		^{1}CT (S_2)		^{3}LE1 (T_1)		^{3}LE2 (T_2)		^{3}CT (T_3)	
S_0	-1568.1779	0.00	-1568.1695	0.23	-1568.1661	0.32	-1568.168	0.27	-1568.1697	0.22	-1568.1654	0.34
^{1}LE (S_1)	-1568.0648	3.08	-1568.0756	2.79	-1568.0642	3.09	-1568.0741	2.83	-1568.0718	2.89	-1568.062	3.16
^{1}CT (S_2)	-1568.0532	3.39	-1568.0549	3.35	-1568.0644	3.09	-1568.0533	3.39	-1568.0536	3.38	-1568.0613	3.17
^{3}LE1 (T_1)	-1568.1035	2.02	-1568.1141	1.74	-1568.103	2.04	-1568.116	1.69	-1568.1098	1.85	-1568.1003	2.11
^{3}LE2 (T_2)	-1568.0664	3.03	-1568.0754	2.79	-1568.0651	3.07	-1568.0711	2.91	-1568.0814	2.63	-1568.063	3.13
^{3}CT (T_3)	-1568.0559	3.32	-1568.0574	3.28	-1568.0679	2.99	-1568.0559	3.32	-1568.0561	3.32	-1568.0652	3.07

Supplementary Table 6 | Energy landscape of MeThPAI. Electronic state energies computed for MeThPAI at the DFT-MRCI/TZVP level on the corresponding PBEh-3c geometries. The TDA-TDDFT formalism has been used for the excited state geometry calculations. The relative energies w.r.t. the S_0 energy at the S_0 geometry are given in electron volts (eV) on the right next to the total energy (in Hartree) of the respective state.

MeThPAI	Minimum geometry											
Electronic state (designation in the main manuscript)	S_0		^{1}LE (S_1)		^{1}CT (S_2)		^{3}LE1 (T_1)		^{3}LE2 (T_2)		^{3}CT (T_3)	
S_0	-1776.8651	0.00	-1776.8535	0.32	-1776.8517	0.37	-1776.8535	0.32	-1776.8534	0.32	-1776.8522	0.35
^{1}LE (S_1)	-1776.7468	3.22	-1776.7595	2.88	-1776.7477	3.20	-1776.7572	2.94	-1776.7563	2.96	-1776.7469	3.22
^{1}CT (S_2)	-1776.7365	3.50	-1776.7384	3.45	-1776.751	3.11	-1776.7363	3.51	-1776.7381	3.46	-1776.7442	3.29
^{3}LE1 (T_1)	-1776.7877	2.11	-1776.7977	1.83	-1776.7883	2.09	-1776.7999	1.77	-1776.7936	1.95	-1776.7856	2.17
^{3}LE2 (T_2)	-1776.7438	3.30	-1776.7573	2.93	-1776.7478	3.19	-1776.7504	3.12	-1776.7652	2.72	-1776.7464	3.23
^{3}CT (T_3)	-1776.7399	3.41	-1776.7412	3.37	-1776.7531	3.05	-1776.7393	3.42	-1776.7404	3.39	-1776.7492	3.16

Supplementary Table 7 | Energy landscape of BeThPAI. Electronic state energies computed for BeThPAI at the DFT-MRCI/TZVP level on the corresponding PBEh-3c geometries. The TDA-TDDFT formalism has been used for the excited state geometry calculations. The relative energies w.r.t. the S_0 energy at the S_0 geometry are given in electron volts (eV) on the right next to the total energy (in Hartree) of the respective state. For BeThPAI, we could not obtain the CT geometries due to state crossings during optimization.

BeThPAI	Minimum geometry									
Electronic state (designation in the main manuscript)	S_0		^{1}LE (S_1)		^{3}LE1 (T_1)		^{3}LE2 (T_2)		^{3}LE3 (T_3)	
S_0	-1891.1692	0.00	-1891.1577	0.31	-1891.158	0.30	-1891.1574	0.32	-1891.1627	0.18
^{1}LE (S_1)	-1891.0503	3.24	-1891.0627	2.90	-1891.0596	2.98	-1891.0586	3.01	-1891.0534	3.15
^{1}CT (S_2)	-1891.0377	3.58	-1891.039	3.54	-1891.0363	3.62	-1891.0382	3.56	-1891.0348	3.66
^{3}LE1 (T_1)	-1891.0908	2.13	-1891.1016	1.84	-1891.04207	1.79	-1891.04207	1.99	-1891.04207	2.08
^{3}LE2 (T_2)	-1891.047	3.33	-1891.0624	2.91	-1891.04207	3.21	-1891.04207	2.69	-1891.04207	3.10
^{3}CT (T_3)	-1891.0414	3.48	-1891.0418	3.47	-1891.04207	3.51	-1891.04207	3.50	-1891.0396	3.53

Supplementary Table 8 | Influence of different minimum geometries. Overview of excited state energies, spin orbit coupling and transfer rates for the T_3 (^{3}CT) → S_1 (^{1}LE) transitions obtained from theoretical investigation based on different geometries. While the Tamm-Dancoff approximated TD-PBEh-3c minimum T_3 (CT) geometry results are each given in the left columns, the S_2 (CT) optimized geometry results are given in the right columns each.

	ΔE_{adia} ($T_3 - S_1$) [eV]		SOC ($T_3 \rightarrow S_1$) [cm^{-1}]		k_{RISC} ($T_3 - S_1$) [s^{-1}]		ΔE_{adia} ($T_3 - T_2$) [eV]	
	triplet CT optimized min. geometry	singlet CT optimized min. geometry	triplet CT optimized min. geometry	singlet CT optimized min. geometry	triplet CT optimized min. geometry	singlet CT optimized min. geometry	triplet CT optimized min. geometry	singlet CT optimized min. geometry
MeFuPAI	0.19	0.18	1.00	1.05	$6.3 \cdot 10^7$	$7.1 \cdot 10^7$	0.29	0.28
BeFuPAI	0.28	0.20	0.38	0.85	$1.2 \cdot 10^7$	$2.4 \cdot 10^7$	0.44	0.36
MeThPAI	0.28	0.17	0.93	1.89	$3.1 \cdot 10^7$	$3.9 \cdot 10^5$	0.44	0.33

Simulation of internal conversion within the triplet state manifold

We find that RISC is more favorable to occur to the ^{1}LE state from the ^{3}CT state than from the ^{3}LE2 state (see Table 2 in the manuscript and Supplementary Table 8). Since the ^{3}CT state is found to be higher in energy at the Franck-Condon point, we want to find out, if population transfer from the ^{3}LE2 to the ^{3}CT state may occur and if it is competitive to the transfer to the energetically lower ^{3}LE1 state. Nonadiabatic dynamics simulations can be employed to elucidate this, but are also computationally elaborate, since many molecular dynamics trajectories need to be calculated.

In this work, we therefore use the ab initio floating occupation molecular orbital-complete active space configuration interaction (FOMO-CASCI) approach[21] in combination with the spherical def2-SV(P)[22,23] basis set as implemented, including gradients[24] and derivative coupling vectors,[25] in the electronic structure program TeraChem.[26] Here, the computationally expensive steps, i.e., the evaluation of two-electron integrals[27,28] and the CASCI calculation[29,30] are accelerated graphics processing units (GPUs). The FOMO-CASCI approach has been suggested as an efficient "one-shot" alternative to state-averaged complete active space self-consistent field (SA-CASSCF) calculations.[21] Different from the latter, the orbitals in FOMO-CASCI are obtained from finite-temperature Hartree-Fock calculations enabling smearing of orbital populations, typically within the same active orbital space that is considered in the CASCI step. The electronic temperature is the key parameter of the method that is generally selected system specifically along with the respective active space. For our purpose, the relative state ordering and energetic splittings of the low-lying triplet states are important, and we find an active space of four electrons in six orbitals together with equal distribution of the active electrons in the active orbital space (i.e., infinite temperature smearing) to reproduce the relevant state orderings well. We refer to this approach as FOMO(∞)-CAS(4,6)CI/def2-SV(P) in the following. We identify this approach to be very well-performing by comparison of the relative energies of the lowest three triplet states to the respective DFT-MRCI/TZVP energies each computed at the TDA-TDDFT-PBEh-3c-optimized geometries. The differences in relative triplet energies between both levels of theory are very small (root mean square deviation of 0.1 eV) and in no case exceed 0.3 eV.

The FOMO(∞)-CAS(4,6)CI/def2-SV(P) electronic energies are furthermore augmented with a London-dispersion correction in the form of the pairwise D3(BJ) model[11,13] (Hartree-Fock parametrization). Nonadiabatic dynamics simulations with this electronic structure approach are performed in the framework of ab initio multiple spawning (AIMS) as implemented in the FMS90 program.[31–33] The AIMS method provides a semi-classical approximation to the nuclear wave packet by means of Gaussian basis functions traveling on a trajectory provided by the Born-Oppenheimer potential energy surface (termed trajectory basis functions or TBFs) of each electronic state. With this approach, we want to study the nonradiative spin-allowed population transfer starting from the ^{3}LE2 state, but restrict this to the MeFuPAI, BeFuPAI, and MeThPAI systems.

To generate the initial conditions for the simulations, the S_0 geometry is first reoptimized at the PBE0-D3(BJ)/def2-TZVP(-f)[11,13,17,23,34,35] level with TeraChem and then the corresponding nuclear Hessian are calculated. Initial conditions, i.e., nuclear positions and velocities, are sampled from a harmonic Wigner

distribution[36] based on this Hessian. We chose the S_0 state minima for generation of the initial conditions, because it best represents the points in phase space that are populated when being directly excited to the ^{3}LE2 state such as in the electroluminescence experiment.

By monitoring the magnitudes of the FOMO(∞)-CAS(4,6)CI/def2-SV(P) state dipole moments, we notice that the ^{3}LE2 (dipole moment < 10 D) is not always lower than the ^{3}CT state (dipole moment > 10 D) on some of the initial condition geometries. Instead a ratio of 57:33:10 (MeFuPAI), 61:27:12 (BeFuPAI), and 23:5:2 (MeThPAI) is observed. Here, the first value gives the counts among the initial condition geometries with ^{3}LE2 being lower than ^{3}CT, the second value indicating counts where the situation is reversed, and the last number corresponding to not clearly distinguishable situations. To estimate the situation in the photoluminescence experiment, we only consider initial conditions where the ^{3}LE2 state is initially the lower one, i.e., the T_2 state. In total, AIMS dynamics simulations based on 10 such initial conditions are carried out for each molecule. The TBFs are propagated using a 20 atomic unit (~0.5 fs) timestep, which is reduced to a timestep of 5 a.u. in regions of high nonadiabatic coupling. Due to the growing computational cost associated with running multiple trajectories after spawning events, we terminate the simulations at 120 fs (MeFuPAI), 90 fs (BeFuPAI), and 20 fs (MeThPAI). In the latter case, more than 60% of the population has decayed to the T_1 (^{3}LE1) state, while for the furanyl systems, nearly all of the population remained on the ^{3}LE2 with few population transfer events occurring mostly to the ^{3}CT state (see Supplementary Figure 9). This is an indication that for these systems, decay to the ^{3}LE1 state is unlikely and the systems can remain on the ^{3}LE2 state for a substantial amount of time and eventually allowing population transfer to occur to the ^{3}CT state from which RISC to the singlet manifold can occur efficiently (see Table 2 in the manuscript).

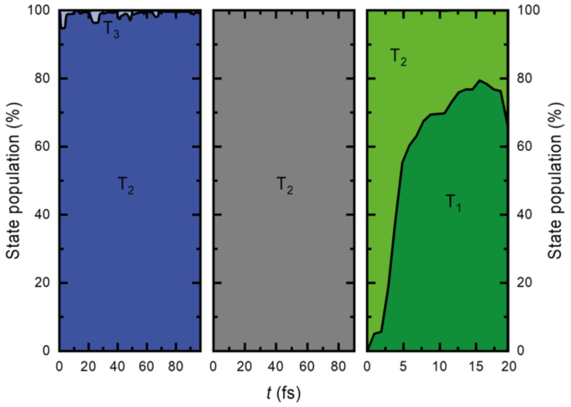

Supplementary Figure 9 | Simulation of optical excitation. Development of the population distribution between T_1, T_2, T_3 where the T_2 state is initially the ^{3}LE2 state. Population vs. time is shown for MeFuPAI (left), BeFuPAI (middle) and MeThPAI (right).

As mentioned above, a substantial number of initial condition geometries shows an inverted state ordering for the furanyl derivatives (37% and 31% for MeFuPAI and BeFuPAI, respectively). Particularly in the setting of an electroluminescence experiment, these initial conditions should also be accessible due to the high excess energy upon excitation. Using 10 of such initial conditions for each of the furanyl derivatives, we perform AIMS simulations for a duration of 15 fs. After this time, 80% of the original ^{3}LE2 population has been transferred to the ^{3}CT state. Hence, these points in phase space allow for increased population transfer to the ^{3}CT state from which we expect the RISC to take place primarily. To obtain an approximated picture for electrical excitation, we use a combination of the previous simulations with the ^{3}CT states having higher energy than the ^{3}LE2 states and the inverted case. We calculate the respective weighted averages according to the distribution of both cases (57:33 for MeFuPAI and 61:27 for BeFuPAI).

To investigate the possible scenario that for MeFuPAI and BeFuPAI, the system might equilibrate tosome extent on the T_2 (^{3}LE2) state, we also consider minimum energy conical intersections (MECI) to. We find for MeFuPAI MECI(T_1/T_2) = 0.50 eV and MECI(T_2/T_3) = 0.32 eV relative to the respective T_2 minimum, and for BeFuPAI MECI(T_1/T_2) = 0.41 eV and MECI(T_2/T_3) = 0.42 eV. This indicates that population will reside on the T_2 for longer than a microsecond. At the same time, up-funneling to T_3 in MeFuPAI appears to be preferred, while a 1:1 ratio of up- (T_3) and down-funneled (T_1) states in

BeFuPAI. These calculations were also performed at the FOMO(∞)-CAS(4,6)CI/def2-SV(P) level in TeraChem.

Due to the small sample size and short time scales, our assessment is, of course, only qualitative. However, taken together the AIMS simulation results, we could collect more support for the hypotheses that, at least for the furanyl derivatives, internal conversion from the ^{3}LE2 to the ^{3}LE1 state is unlikely and instead, population transfer to the ^{3}CT can take place.

Supplementary Table 9 | Minimum energy conical intersections. Electronic energies (in Hartree) for the T_2 minima and MECIs computed at the FOMO(∞)-CAS(4,6)CI/def2-SV(P) level (including D3(BJ) dispersion energies. Relative energies in eV are given in parentheses.

	T_2 minimum	T_1/T_2 MECI	T_2/T_3 MECI
MeFuPAI	-1444.34161	-1444.32328 (0.50 eV)	-1444.32993 (0.32 eV)
BeFuPAI	-1557.91861	-1557.90355 (0.41 eV)	-1557.90334 (0.42 eV)

Supplementary Note 3: Electroluminescence

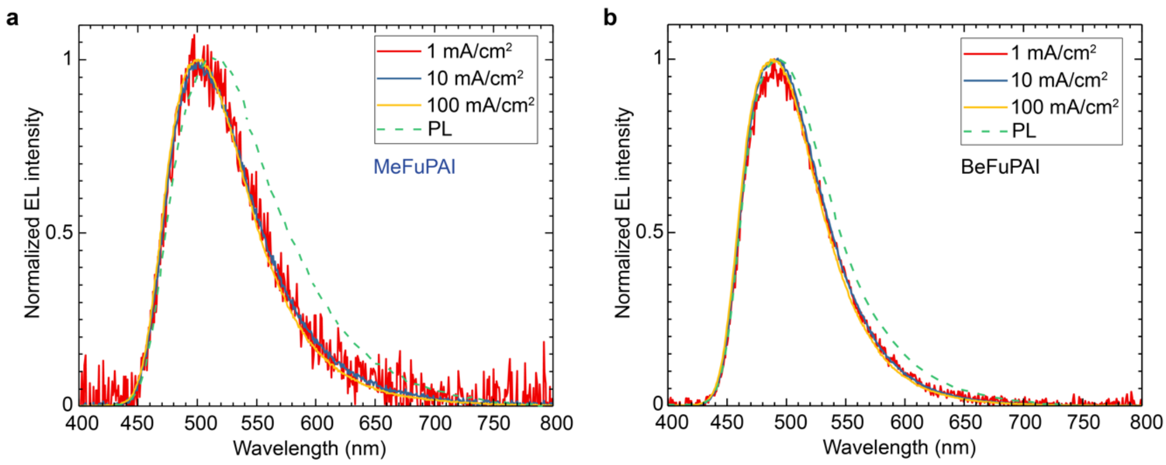

Supplementary Figure 10 | Luminescence spectra. Photoluminescence and electroluminescence spectra of MeFuPAI (a) and BeFuPAI (b) OLEDs at different current densities.

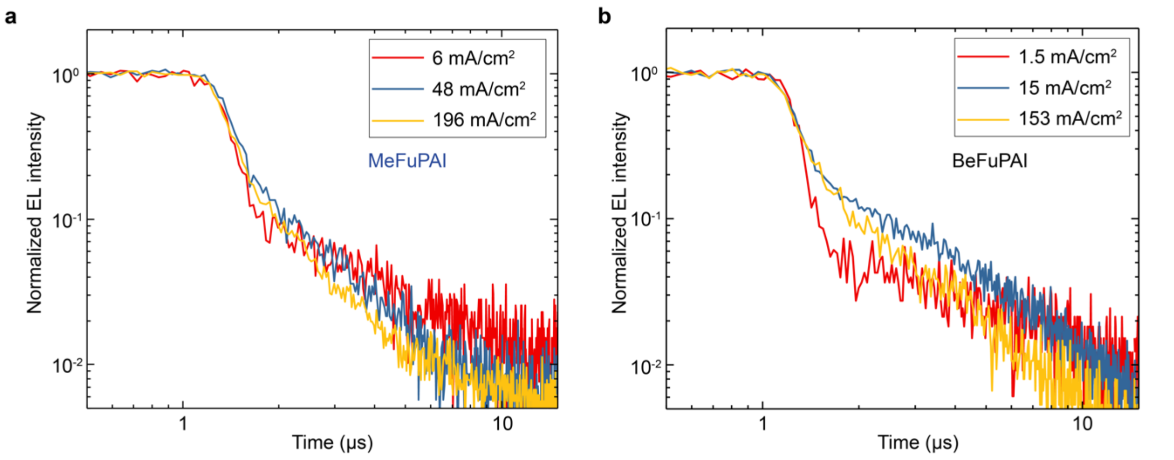

Supplementary Figure 11 | Transient electroluminescence. Transient electroluminescence spectra of MeFuPAI (a) and BeFuPAI (b) OLEDs at different current densities.

Supplementary Note 4: Calculation of I_{HE}

The following formula gives the ratio of emission intensity of populated triplet to singlet states, which often is synonymous with the intensity-ratio of the prompt and delayed emission components.[37]

$$\frac{I_\mathrm{T}}{I_\mathrm{S}} = 4\eta_\mathrm{r} - 1$$

However, in our case, hot exciplex emission (I_HE) originating from triplets can be assigned to the prompt component:

$$I_\mathrm{S} + I_\mathrm{HE} = I_\mathrm{pr}$$
$$I_\mathrm{T} = I_\mathrm{HE} + I_\mathrm{TTA\text{-}UC}$$
$$I_\mathrm{TTA\text{-}UC} = I_\mathrm{del}$$

Inserting into the previous equation yields an expression for I_HE:

$$I_\mathrm{HE} = \frac{I_\mathrm{pr}(4\eta_\mathrm{r} - 1)}{4\eta_\mathrm{r}} - \frac{I_\mathrm{del}}{4\eta_r}$$

Normalizing the delayed and prompt components to unity:

$$I_\mathrm{del} = 1 - I_\mathrm{pr}$$

yields the simplified equation:

$$I_{\mathrm{HE}} = I_{\mathrm{pr}} - \frac{1}{4\eta_{\mathrm{r}}}$$

Normalizing the equation for transient EL analysis to unity, as well, allows expression of I_{pr} depending only on η_r (subscript *transEL* added for clarification of the η_r origin):

$$I_{\mathrm{pr}} = \frac{1}{4\eta_{\mathrm{r,transEL}}}$$

Inserting into the previous, simplified equation for I_{HE} yields the final equation (subscript *EQE* added for clarification of the η_r origin):

$$I_{\mathrm{HE}} = \frac{1}{4\eta_{\mathrm{r,\ transEL}}} - \frac{1}{4\eta_{\mathrm{r,\ EQE}}}$$

Supplementary Note 5: Experimentals

Materials

All reagents and solvents were purchased from commercial suppliers (abcr, TCI, Sigma Aldrich, VWR) and used without further purification.

Synthetic Procedures

1,8-Dibromoanthraquinone (**2**)

Under stirring with a mechanical stirrer, a suspension of **1** (150 g, 541 mmol), KBr (300 g, 2.52 mol), and $CuCl_2$ (20.0 g, 149 mmol) in nitrobenzene (1 L) and phosphoric acid (85 wt% in H_2O, 200 mL) is heated up to 200 °C. After all water has been evaporated and the reaction temperature has been reached, the reaction is stirred for 36 h at 200 °C. The dark green suspension is poured into methanol (5L). The resulting brown precipitate is isolated by filtration and washed with methanol (500 mL) and water (1L) to remove generated KCl. The crude product is re-dissolved in nitrobenzene (738 ml) and phosphoric acid (148 mL), and KBr (222 g, 1.86 mol) and $CuCl_2$ (14.5 g, 108 mmol) is added. The reaction mixture is stirred at 200 °C for 20 h and then worked up as before, to give 1,8-dibromanthraquinon (**2**, 109 g, 298 mmol, 55 %) as brown solid. ^{1}H-NMR (400 MHz, $CDCl_3$): δ [ppm] = 8.25 (dd, $^3J_{H,H}$ = 7.8 Hz, $^4J_{H,H}$ = 1.2 Hz, 2H), 8.03 (dd, $^3J_{H,H}$ = 7.9 Hz, $^4J_{H,H}$ = 1.2 Hz, 2H), 7.55 (dd, $^3J_{H,H}$ = 7.9 Hz, $^3J_{H,H}$ = 7.8 Hz, 2H). APCI-MS (pos.): [M] = $C_{14}H_6Br_2O_2$, experimental m/z = 366.8795 (found [M+H$^+$]), calculated m/z = 366.8792.

1,8-Dibromo-10-anthrone (**3**)

Under stirring at -78 °C, $NaBH_4$ (41.4 g, 1.09 mol) is added in portions to a solution of **2** (50.0 g, 137 mmol) in methanol (1.5 L). The reaction is kept at this temperature for three hours, and then concentrated HCl (150 mL) is added, and the reaction mixture is stirred for 16 hours under reflux. The resulting precipitate is filtered, washed with water (500 mL), dissolved in methanol (1.5 L), and cooled to -78 °C. $NaBH_4$ (41.4 g, 1.09 mol) is added and the reaction mixture is stirred for 2 hours at -78 °C. Then, concentrated HCl (150 mL) is added and the reaction mixture is refluxed for 1 h. The precipitate is filtered, washed with water (500 mL) and purified by column chromatography (SiO_2, hexane/DCM 10:1 → 1:1) to give **3** (20.9 g, 59.4 mmol, 43 %) as yellow solid. ^{1}H-NMR (400 MHz, $CDCl_3$): δ [ppm] = 8.34 (dd, $^3J_{H,H}$ = 7.9 Hz, $^4J_{H,H}$ = 1.3 Hz, 2H), 7.90 (dd, $^3J_{H,H}$ = 7.8 Hz, $^4J_{H,H}$ = 1.3 Hz, 2H), 7.40 (dd, $^3J_{H,H}$ = 7.9 Hz, $^3J_{H,H}$ = 7.8 Hz, 2H), 4.19 (s, 2H). MALDI-MS (DCTB, pos.): [M] = $C_{14}H_8Br_2O$, experimental m/z = 351.8915 (found [M$^+$]), calculated m/z = 351.8921.

1,8-Dibromo-10-phenylanthracene (**4**)

Under argon, a solution of **3** (20.0 g, 53.8 mmol) in anhydrous toluene (200 mL) is prepared in a heated Schlenk flask and cooled to -78 °C. A solution of phenyl lithium (1.9 M in dibutyl ether, 29.9 mL, 56.8 mmol) is added dropwise under stirring, and the reaction mixture is allowed to slowly warm up to

room temperature. After stirring for 20 h, concentrated HCl (50 mL) is added, and the reaction mixture is stirred under reflux for 3 h. The organic material is extracted with toluene (3x 200 mL) and dried over Na_2SO_4. After the solvent is removed under reduced pressure, the crude product is purified by column chromatography (SiO_2, hexane) to give **4** (16.2 g, 39.1 mmol, 69 %) as yellow solid. ^{1}H-NMR (400 MHz, $CDCl_3$): δ [ppm] = 9.37 (s, 1H), 7.85 (d, $^3J_{H,H}$ = 7.1 Hz, 2H), 7.62-7.55 (m, 5H), 7.39-7.36 (m, 2H) 7.21 (dd, $^3J_{H,H}$ = 8.8 Hz, $^3J_{H,H}$ = 7.1 Hz, 2H). APCI-MS (pos.): [M] = $C_{20}H_{12}Br_2$, experimental m/z = 411.9299 (found [M^+]), calculated m/z = 411.9285.

General procedure for Suzuki coupling reactions

A sealed vial equipped with a magnetic stirring bar, K_3PO_4 (2.0 eq), $Pd_2(dba)_3$ (0.015 eq), P(*o*-tol)$_3$ (0.12 eq), anthracene compound **4-8** (1.0 eq), and the boronic acid (pinacol ester) (1.0/1.05 eq) is evacuated and charged with argon three times. Then, a solvent mixture from water, 1,4-dioxane, and toluene (1/5.5/1.8) is added, and the reaction mixture is degassed by bubbling with argon for 10 minutes. The reaction is stirred at 60 °C for 24 h and subsequently diluted with water. The diluted mixture is extracted with chloroform (3x 25 mL), and the combined organic extracts are dried over Na_2SO_4. After the solvent has been removed under reduced pressure, the crude product is purified *via* column chromatography over silica to obtain the desired compound.

2-(8-Bromo-10-phenylanthracen-1-yl)-5-methylfuran (**5**)

The reaction between K_3PO_4 (1.29 g, 6.07 mmol), $Pd_2(dba)_3$ (41.7 mg, 45.5 μmol), P(*o*-tol)$_3$ (111 mg, 364 μmol), **4** (1.25 g, 3.03 mmol), and 5-methylfuran-2-boronic acid pinacol ester (619 mg, 3.03 mmol) is carried out in 60 mL solvent according to the general procedure. After column chromatography (SiO_2, hexane/DCM 1:0 → 9:1), **5** (480 mg, 1.16 mmol, 38 %) is obtained as yellow solid. ^{1}H-NMR (400 MHz, $CDCl_3$): δ [ppm] = 9.65 (s, 1H), 7.80-7.78 (m, 2H), 7.61-7.54 (m, 5H), 7.42-7.40 (m, 3H), 7.17 (dd, $^3J_{H,H}$ = 8.8 Hz, $^3J_{H,H}$ = 7.1 Hz, 1H), 6.90 (d, $^3J_{H,H}$ = 3.1 Hz, 1H), 6.29-6.28 (m, 1H), 2.52 (s, 3H). MALDI-MS (pos.): [M] = $C_{25}H_{17}Br_2O$, experimental m/z = 412.0451 (found [M(^{79}Br)$^+$]), calculated m/z = 412.0463.

2-(8-Bromo-10-phenylanthracen-1-yl)benzofuran (**6**)

The reaction between K_3PO_4 (1.29 g, 6.07 mmol), $Pd_2(dba)_3$ (41.7 mg, 45.5 μmol), P(*o*-tol)$_3$ (111 mg, 364 μmol), **4** (1.25 g, 3.03 mmol), and 2-benzofuranylboronic acid (491 mg, 3.03 mmol) is carried out in 60 mL solvent according to the general procedure. After column chromatography (SiO_2, hexane/DCM 1:0 → 9:1), **5** (471 mg, 1.05 mmol, 35 %) is obtained as yellow oil. ^{1}H-NMR (400 MHz, $CDCl_3$): δ [ppm] = 9.70 (s, 1H), 8.00 (d, 1H), 7.82 (d, $^3J_{H,H}$ = 7.1 Hz, 1H), 7.76 (d, $^3J_{H,H}$ = 7.6 Hz, 1H), 7.71 (d, $^3J_{H,H}$ = 8.9 Hz, 1H), 7.67 (d, $^3J_{H,H}$ = 8.0 Hz, 1H), 7.64-7.56 (m, 4H), 7.47 (dd, $^3J_{H,H}$ = 8.9 Hz, $^3J_{H,H}$ = 6.9 Hz 1H), 7.44-7.42 (m, 2H), 7.40-7.32 (m, 3H), 7.20 (dd, $^3J_{H,H}$ = 8.8 Hz, $^3J_{H,H}$ = 7.1 Hz, 1H). MALDI-MS (pos.): [M] = $C_{28}H_{17}BrO$, experimental m/z = 450.0435 (found [M(^{81}Br)$^+$]), calculated m/z = 450.04423.

2-(8-Bromo-10-phenylanthracen-1-yl)-5-methylthiophene (**7**)

The reaction between K_3PO_4 (1.29 g, 6.07 mmol), $Pd_2(dba)_3$ (41.7 mg, 45.5 μmol), P(*o*-tol)$_3$ (111 mg, 364 μmol), **4** (1.25 g, 3.03 mmol), and 5-methylthiophene-2-boronic acid pinacol ester (668 mg, 3.03 mmol) is carried out in 60 mL solvent according to the general procedure. After column chromatography (SiO_2, hexane/DCM 1:0 → 9:1), **7** (520 mg, 1.21 mmol, 40 %) is obtained as yellow solid. ^{1}H-NMR (400 MHz, $CDCl_3$): δ [ppm] = 9.50 (s, 1H), 7.77 (d, $^3J_{H,H}$ = 7.1 Hz, 1H), 7.62-7.55 (m, 6H), 7.42-7.36 (m, 3H), 7.28 (d, $^3J_{H,H}$ = 3.4 Hz, 1H), 7.16 (dd, $^3J_{H,H}$ = 8.8 Hz, $^3J_{H,H}$ = 7.1 Hz, 1H), 6.95-6.94 (m, 1H), 2.63 (s, 3H). MALDI-MS (pos.): [M] = $C_{25}H_{17}BrS$, experimental m/z = 428.0222 (found [M(^{79}Br)$^+$]), calculated m/z = 428.0234.

2-(8-Bromo-10-phenylanthracen-1-yl)benzo[*b*]thiophene (**8**)

The reaction between K_3PO_4 (1.29 g, 6.07 mmol), $Pd_2(dba)_3$ (41.7 mg, 45.5 μmol), P(*o*-tol)$_3$ (111 mg, 364 μmol), **4** (1.25 g, 3.03 mmol), and benzo[*b*]thien-2-ylboronic acid (540 mg, 3.03 mmol) is carried out in 60 mL solvent according to the general procedure. After column chromatography (SiO_2,

hexane/DCM 1:0 → 9:1), **8** (151 mg, 324 µmol, 11 %) is obtained as yellow solid. ^{1}H-NMR (400 MHz, $CDCl_3$): δ [ppm] = 9.55 (s, 1H), 7.96 (dd, $^3J_{H,H}$ = 7.3 Hz, $^4J_{H,H}$ = 3.6 Hz, 2H), 7.79 (d, $^3J_{H,H}$ = 7.1 Hz, 1H), 7.72-7.69 (m, 3H), 7.64-7.57 (m, 4H), 7.49-7.43 (m, 5H), 7.18 (dd, $^3J_{H,H}$ = 8.9 Hz, $^3J_{H,H}$ = 7.1 Hz 1H). MALDI-MS (pos.): [M] = $C_{28}H_{17}BrS$, experimental m/z = 464.0226 (found [M(^{79}Br)$^+$]), calculated m/z = 464.0234.

5-(8-(5-Methylfuran-2-yl)-10-phenylanthracen-1-yl)isophthalonitrile (MeFuPAI)

The reaction between K_3PO_4 (493 mg, 2.32 mmol), $Pd_2(dba)_3$ (16.0 mg, 17.4 µmol), P(*o*-tol)$_3$ (42.4 mg, 139 µmol), **5** (480 mg, 1.16 mmol), and 5-(4,4,5,5-tetramethyl-1,3,2- dioxaborolan-2-yl)isophthalonitrile (310 mg, 1.22 mmol) is carried out in 50 mL solvent according to the general procedure. After column chromatography (SiO_2, hexane/DCM 1:1 → 0:1), MeFuPAI (377 mg, 819 µmol, 71 %) is obtained as yellow solid. ^{1}H-NMR (400 MHz, $CDCl_3$): δ [ppm] = 8.90 (s, 1H), 8.14 (d, $^4J_{H,H}$ = 1.6 Hz, 2H), 8.05 (t, $^4J_{H,H}$ = 1.6 Hz, 1H), 7.76 (d, $^3J_{H,H}$ = 8.8 Hz, 1H), 7.71 (d, $^3J_{H,H}$ = 6.7 Hz, 1H), 7.64-7.58 (m, 4H), 7.46-7.36 (m, 5H), 6.50 (d, $^3J_{H,H}$ = 3.2 Hz, 1H), 6.19-6.18 (m, 1H), 2.35 (s, 3H). MALDI-MS (pos.): [M] = $C_{33}H_{20}N_2O$, experimental m/z = 460.1562 (found [M$^+$]), calculated m/z = 460.1576.

5-(8-(Benzofuran-2-yl)-10-phenylanthracen-1-yl)isophthalonitrile (BeFuPAI)

The reaction between K_3PO_4 (445 mg, 2.10 mmol), $Pd_2(dba)_3$ (9.6 mg, 10.5 µmol), P(*o*-tol)$_3$ (38.3 mg, 126 µmol), **6** (471 mg, 1.05 mmol), and 5-(4,4,5,5-tetramethyl-1,3,2- dioxaborolan-2-yl)isophthalonitrile (280 mg, 1.10 mmol) is carried out in 50 mL solvent according to the general procedure. After column chromatography (SiO_2, hexane/DCM 1:1 → 0:1), BeFuPAI (379 mg, 763 µmol, 73 %) is obtained as yellow solid. ^{1}H-NMR (400 MHz, $CDCl_3$): δ [ppm] = 8.86 (s, 1H), 8.15 (d, $^4J_{H,H}$ = 1.5 Hz, 2H), 7.96 (t, $^4J_{H,H}$ = 1.5 Hz, 1H), 7.90-7.88 (m, 1H), 7.80-7.74 (m, 3H), 7.66-7.56 (m, 4H), 7.49-7.43 (m, 4H), 7.41-7.31 (m, 3H), 6.99 (s, 1H). MALDI-MS (DCTB, pos.): [M] = $C_{36}H_{20}N_2O$, experimental m/z = 496.1567 (found [M$^+$]), calculated m/z = 496.1576.

5-(8-(5-Methylthiophen-2-yl)-10-phenylanthracen-1-yl)isophthalonitrile (MeThPAI)

The reaction between K_3PO_4 (583 mg, 2.75 mmol), $Pd_2(dba)_3$ (18.9 mg, 17.4 µmol), P(*o*-tol)$_3$ (50.2 mg, 165 µmol), **7** (590 mg, 1.37 mmol), and 5-(4,4,5,5-tetramethyl-1,3,2- dioxaborolan-2-yl)isophthalonitrile (367 mg, 1.44 mmol) is carried out in 50 mL solvent according to the general procedure. After column chromatography (SiO_2, hexane/DCM 1:1 → 0:1), MeThPAI (430 mg, 902 µmol, 66 %) is obtained as yellow solid. ^{1}H-NMR (400 MHz, $CDCl_3$): δ [ppm] = 8.81 (s, 1H), 8.14 (d, $^4J_{H,H}$ = 1.5 Hz, 2H), 8.00 (t, $^4J_{H,H}$ = 1.5 Hz, 1H), 7.77 (d, $^3J_{H,H}$ = 8.3 Hz, 1H), 7.67-7.55 (m, 5H), 7.46-7.38 (5H), 7.02 (d, $^3J_{H,H}$ = 3.4 Hz, 1H), 6.87-6.86 (m, 1H), 2.62 (s, 3H). MALDI-MS (DCTB, pos.): [M] = $C_{33}H_{20}N_2S$, experimental m/z = 476.1333 (found [M$^+$]), calculated m/z = 476.1347.

5-(8-(Benzo[*b*]thiophen-2-yl)-10-phenylanthracen-1-yl)isophthalonitrile (BeThPAI)

The reaction between K_3PO_4 (138 mg, 649 µmol), $Pd_2(dba)_3$ (4.5 mg, 4.9 µmol), P(*o*-tol)$_3$ (11.9 mg, 38.9 µmol), **8** (151 mg, 324 µmol), and 5-(4,4,5,5-tetramethyl-1,3,2- dioxaborolan-2-yl)isophthalonitrile (86.6 mg, 341 µmol) is carried out in 15 mL solvent according to the general procedure. After column chromatography (SiO_2, hexane/DCM 1:1 → 0:1), BeThPAI (82.2 mg, 160 µmol, 49 %) is obtained as yellow solid. ^{1}H-NMR (400 MHz, $CDCl_3$): δ [ppm] = 8.80 (s, 1H), 8.13 (d, $^4J_{H,H}$ = 1.6 Hz, 2H), 8.09-8.07 (m, 1H), 7.90-7.88 (m, 1H), 7.85 (t, $^4J_{H,H}$ = 1.5 Hz, 1H), 7.79 (d, $^3J_{H,H}$ = 8.6 Hz, 1H) 7.74 (d, $^3J_{H,H}$ = 8.8 Hz, 1H), 7.69 (dd, $^3J_{H,H}$ = 8.6 Hz, $^4J_{H,H}$ = 1.2 Hz, 1H), 7.66-7.60 (m, 3H), 7.48-7.39 (m, 8H). MALDI-MS (DCTB, pos.): [M] = $C_{36}H_{20}N_2S$, experimental m/z = 512.1339 (found [M$^+$]), calculated m/z = 512.1347.

Optical Spectroscopy

UV/Vis absorption spectroscopy was carried out on a PerkinElmer Lambda 365 Spectrophotometer. Photoluminescence spectra were recorded on a PerkinElmer FL 6500 Fluorescence Spectrophotometer. Time correlated single photon counting (TCSPC) experiments were conducted between 411 nm and 693 nm on a setup containing a BDS-375-SM-FBE picosecond diode laser (375 nm excitation

wavelength) and a PMC-100-4 photomultiplier tube (both from Becker&Hickl), a sCMOS Zyla sensor, and a SR-303i-B spectrograph (both from Andor Technology). Temperature dependent photoluminescence characteristics were measured using Quantaurus-Tau (Hamamatsu Photonics, C11367) and a cryostat (Oxford Instruments, Optistat DN2).

Device Fabrication

OLEDs were fabricated by vacuum deposition on ITO-coated glass substrates under a pressure of less than 5.0×10^{-4} Pa. Organic layers were deposited at a deposition rate of 0.05 nm/s through a metal mask. After deposition, the metal mask was replaced with another metal mask for cathode deposition in a nitrogen-filled glove box. The 8-quinolinolato lithium (Liq) and Al electrodes were deposited on the organic layers at deposition rates of 0.02 nm/s for Liq and 0.05–0.2 nm/s for Al. The device structure is ITO (100 nm)/HAT-CN (10 nm)/Tris-PCz (30 nm)/PAI (20 nm)/TmPyPB (40 nm)/Liq (4 nm)/Al (70 nm), where HAT-CN is hexaazatriphenylenehexacarbonitrile, Tris-PCz is 9,9′-diphenyl-6-(9-phenyl-9H-carbazol-3-yl)-9H,9′H-3,3′-bicarbazole, and TmPyPB is 1,3,5-tri[(3-pyridyl)-phen-3-yl]benzene.

Electroluminescence spectroscopy

EL spectra, and external EL quantum efficiency–current density characteristics were measured using an absolute EQE measurement system (C9920-12, Hamamatsu Photonics, Japan). Transient EL characteristics under pulse excitation with a pulse width of 100 µs were measured using a streak camera (C4334, Hamamatsu Photonics, Japan).

Supplementary Note 6: NMR spectra

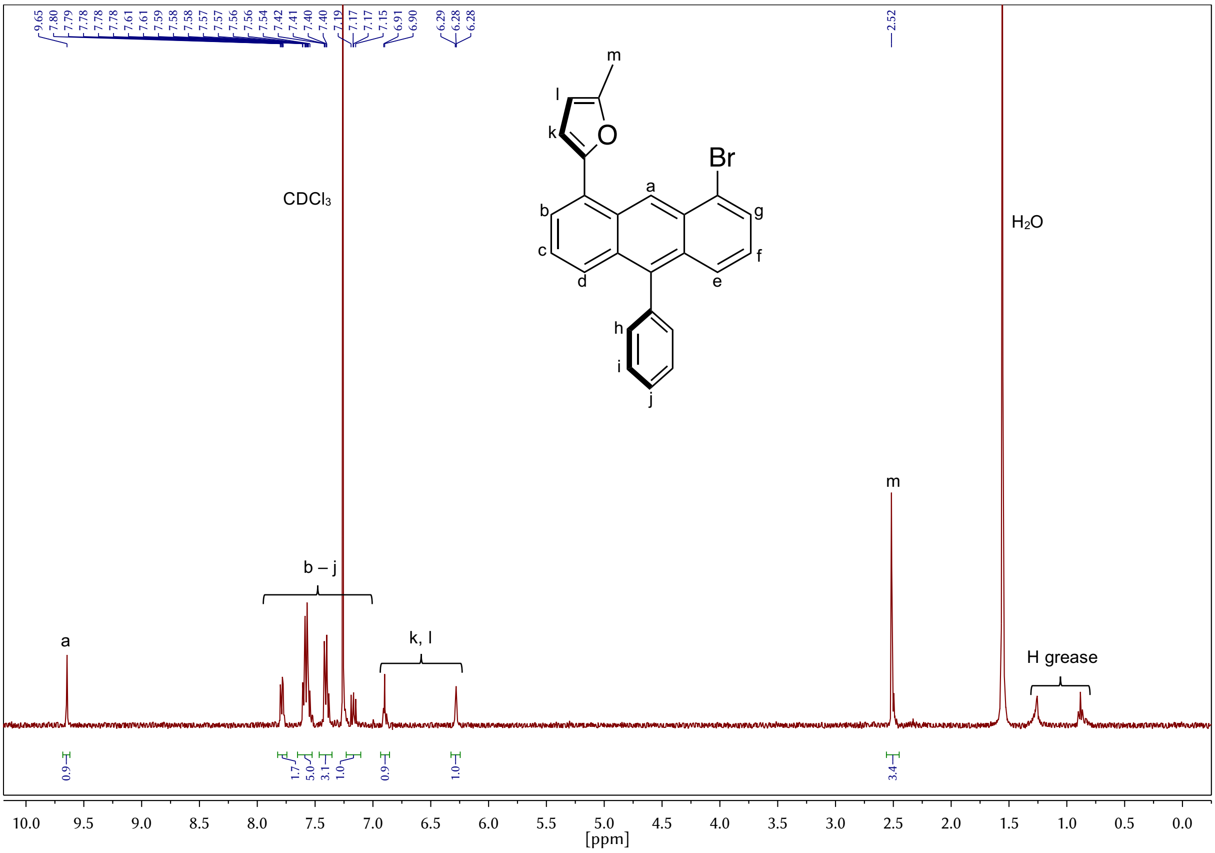

Supplementary Figure 12 | ^{1}H-NMR spectra of 5 in $CDCl_3$.

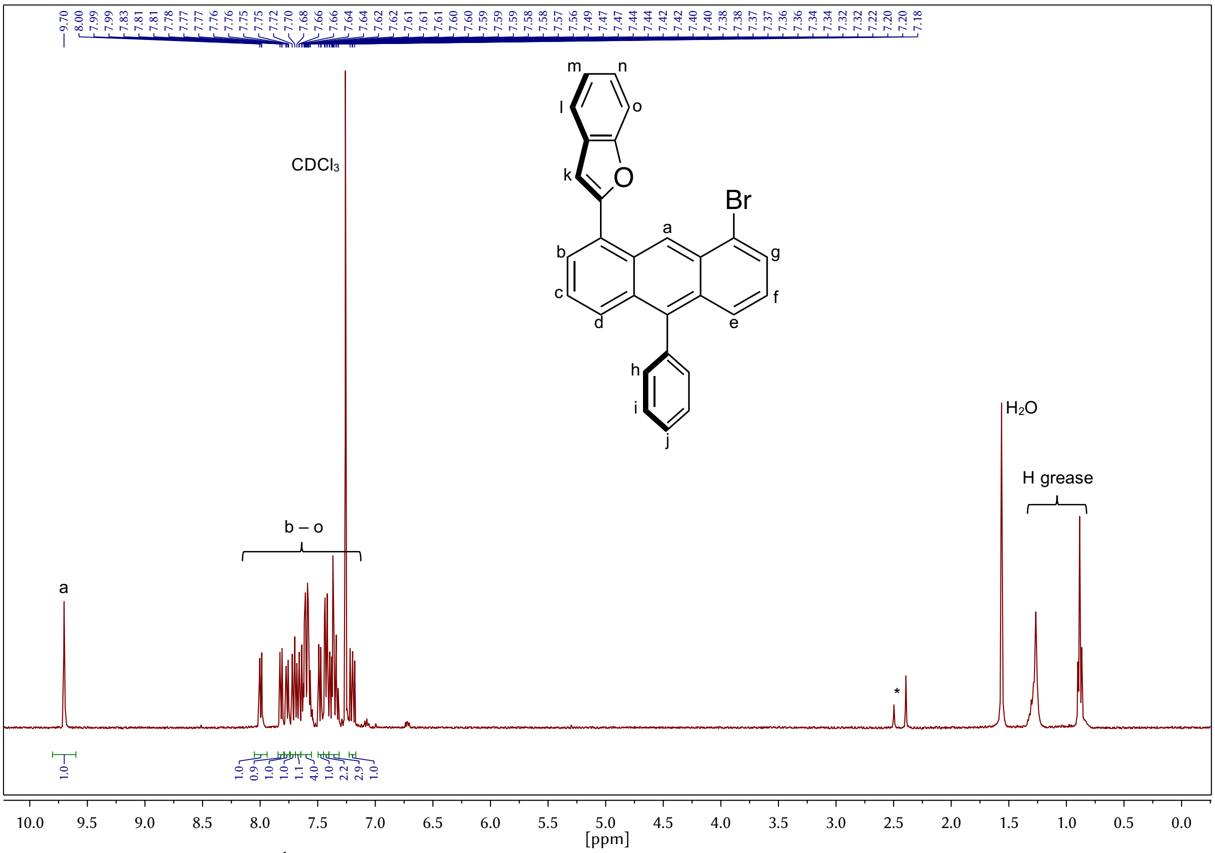

Supplementary Figure 13 | ^{1}H-NMR spectra of 6 in $CDCl_3$. The compound can be used for the subsequent reaction, despite minor impurities (*).

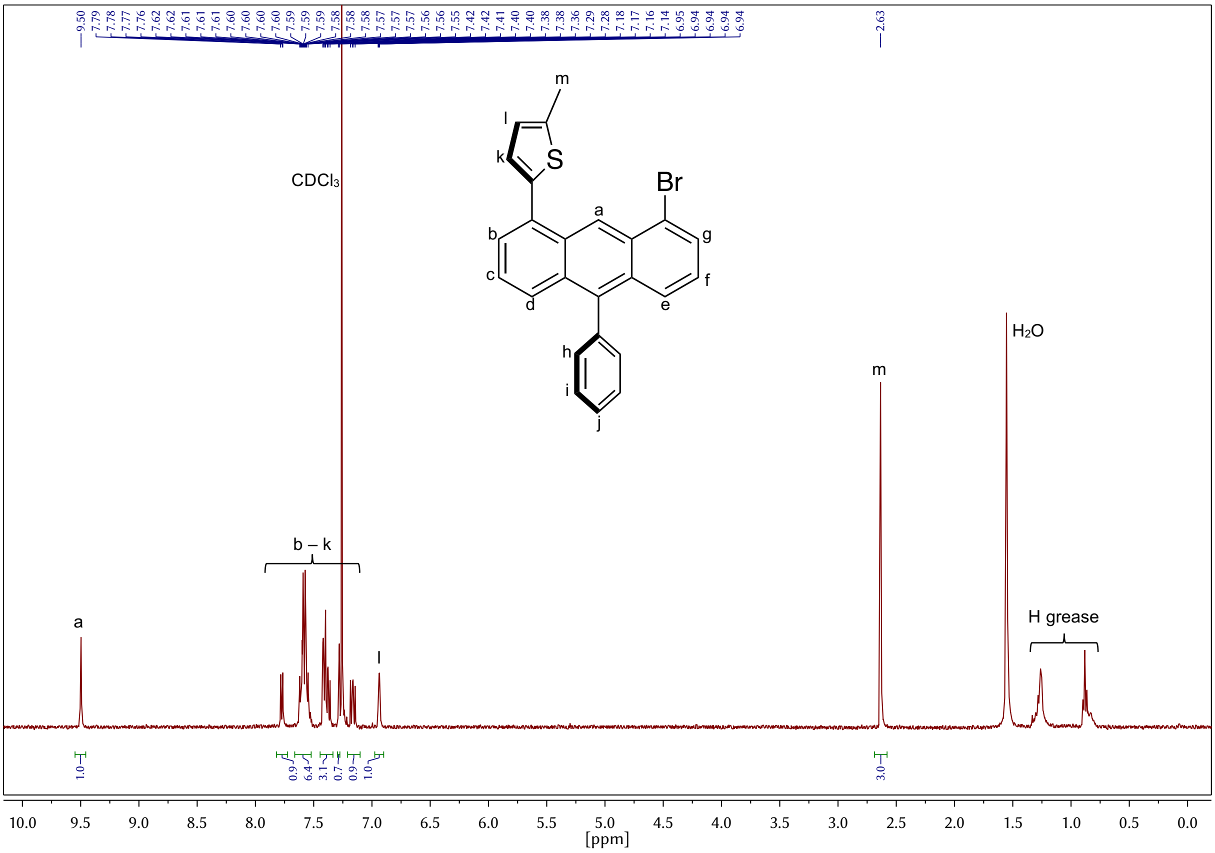

Supplementary Figure 14 | ^{1}H-NMR spectra of 7 in $CDCl_3$.

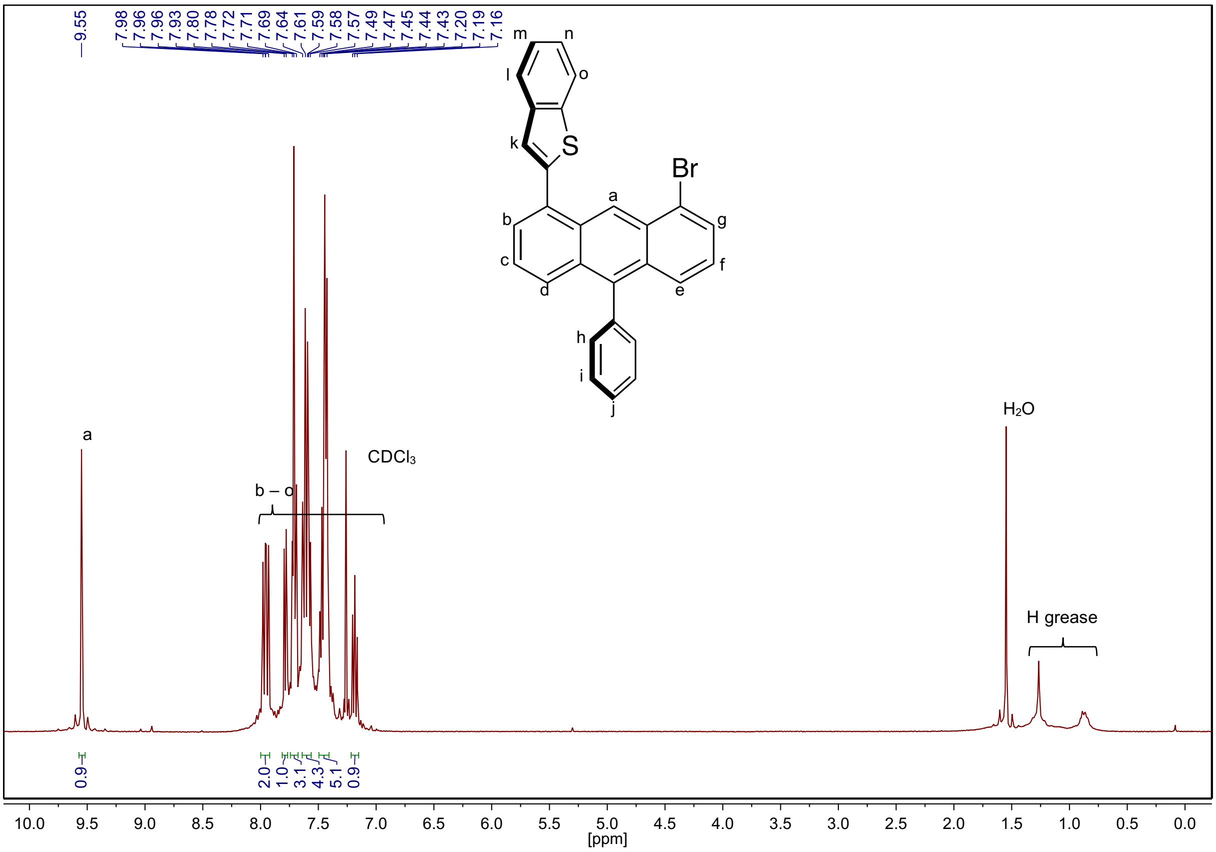

Supplementary Figure 15 | ^{1}H-NMR spectra of 8 in $CDCl_3$.

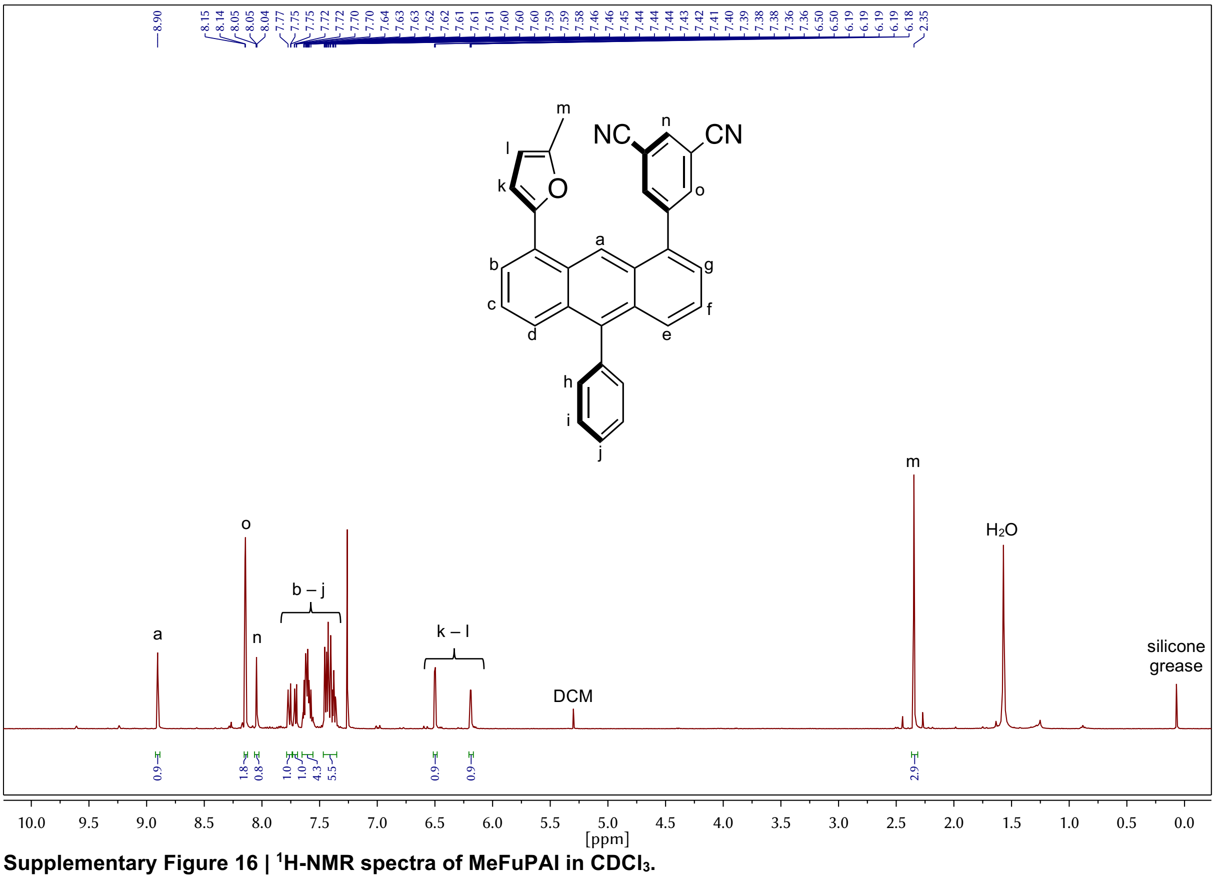

Supplementary Figure 16 | ^{1}H-NMR spectra of MeFuPAI in $CDCl_3$.

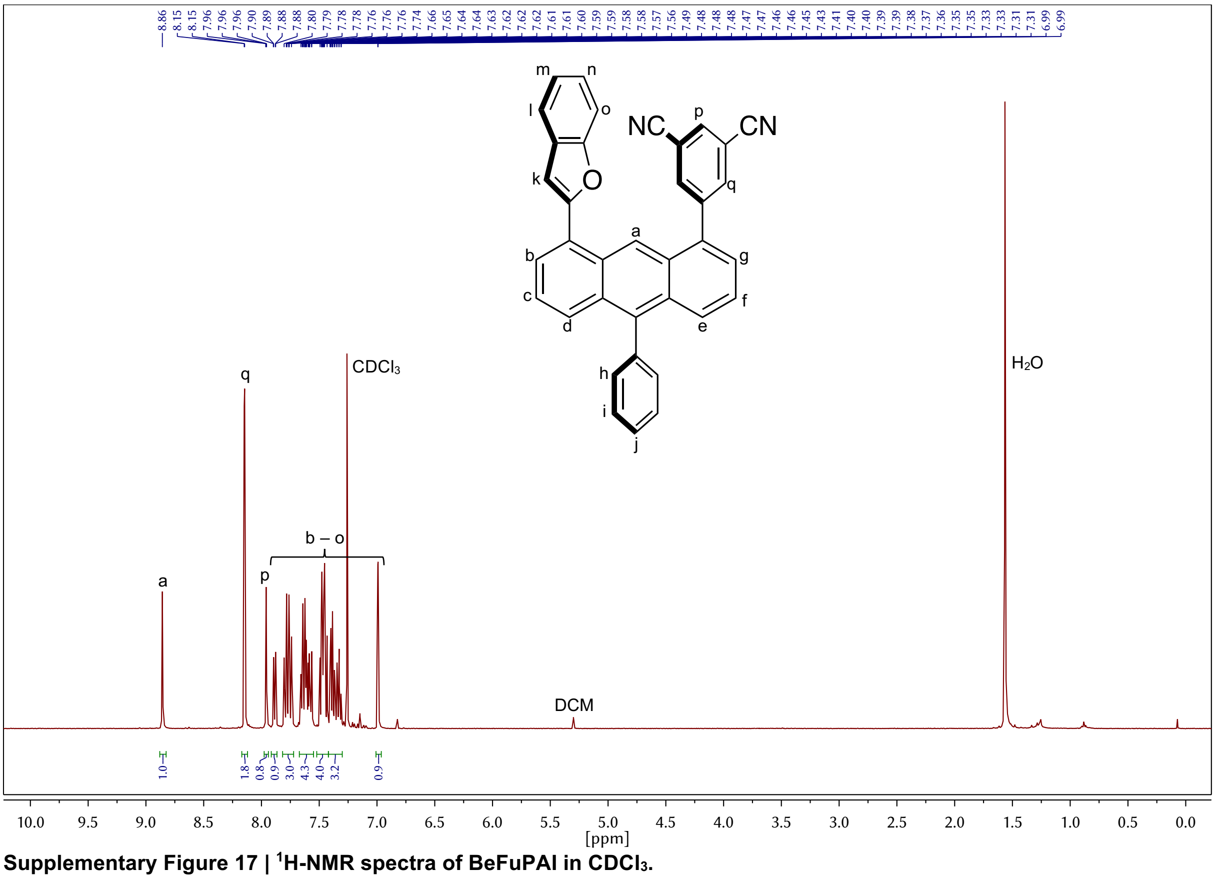

Supplementary Figure 17 | ^{1}H-NMR spectra of BeFuPAI in $CDCl_3$.

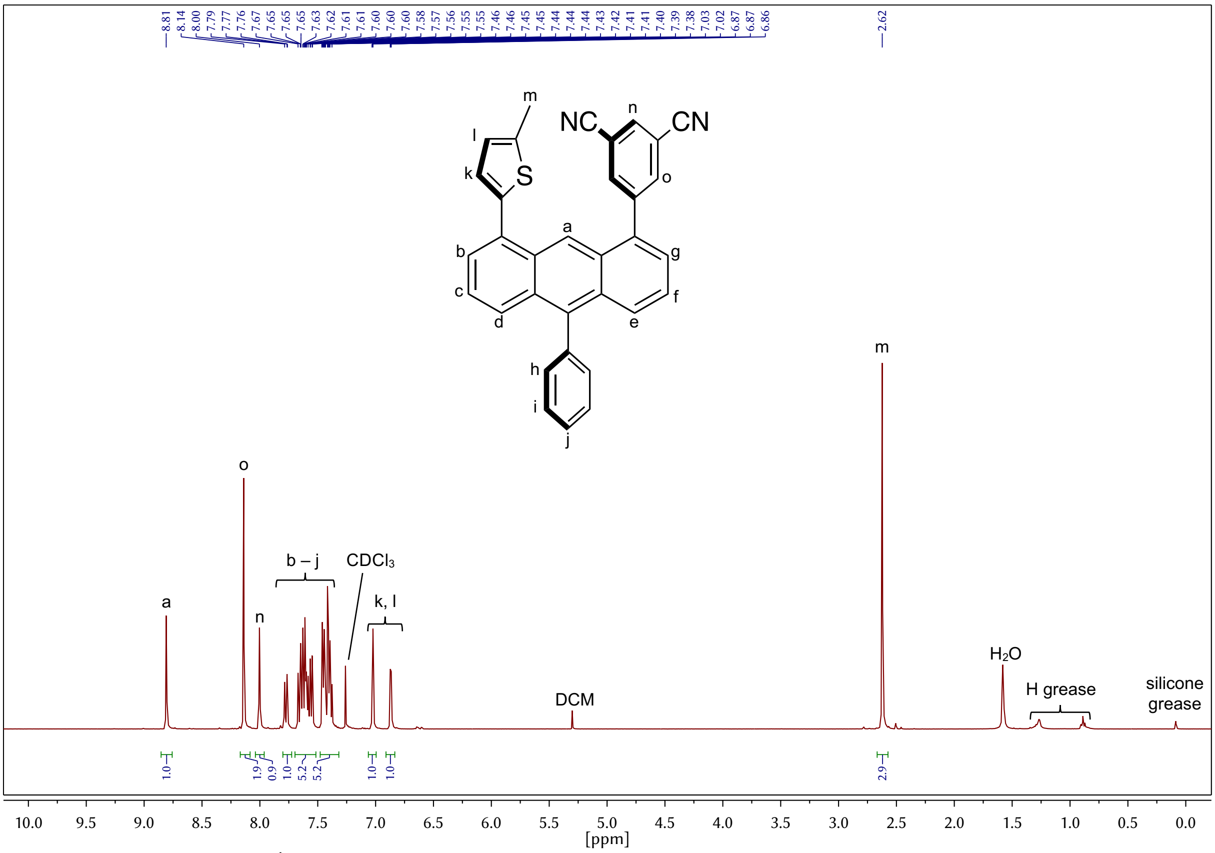

Supplementary Figure 18 | ^{1}H-NMR spectra of MeThPAI in $CDCl_3$.

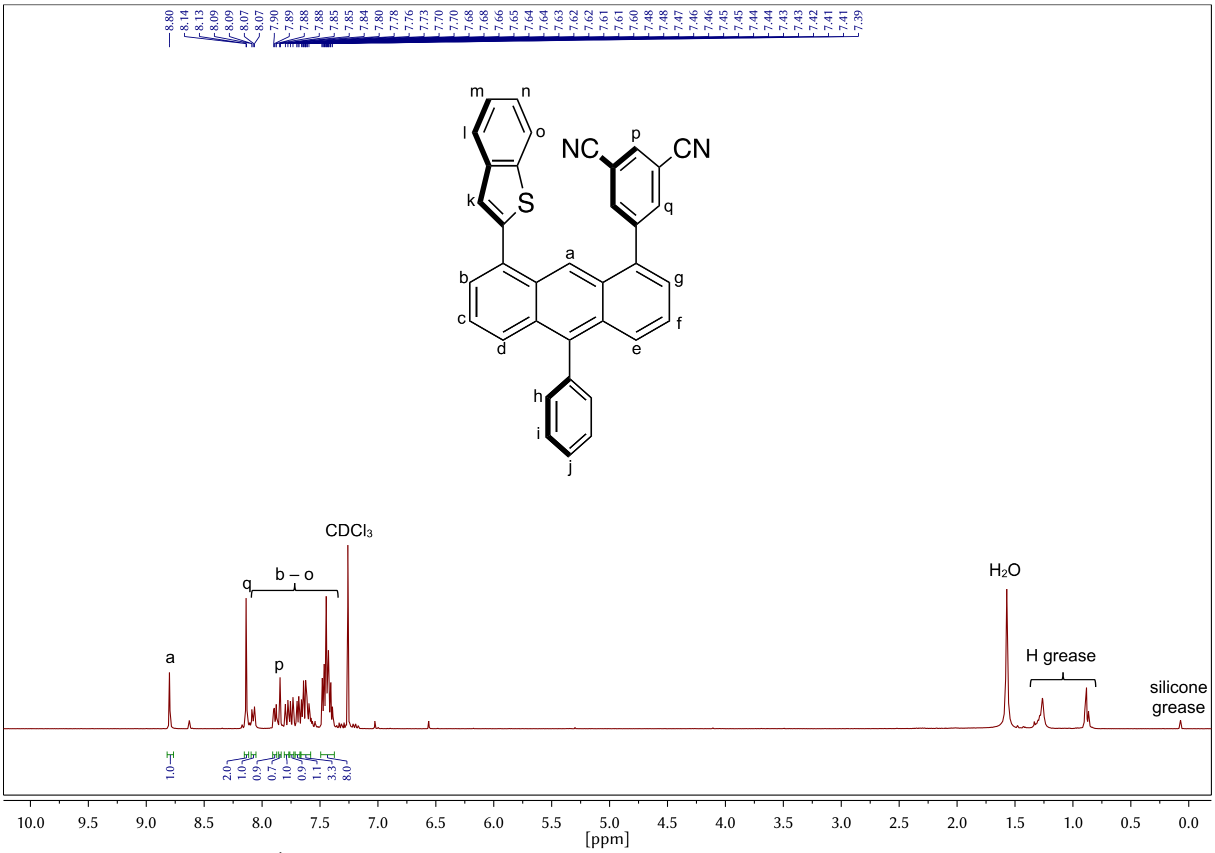

Supplementary Figure 19 | ^{1}H-NMR spectra of BeThPAI in $CDCl_3$.

Supplementary References

1. Frisch, M. J. *et al*. Gaussian 09, Revision D.01. (2016).
2. Tsuchiya, Y. *et al*. Exact Solution of Kinetic Analysis for Thermally Activated Delayed Fluorescence Materials. *J. Phys. Chem. A* accepted (2021) doi:10.1021/acs.jpca.1c04056.
3. Dias, F. B., Penfold, T. J. & Monkman, A. P. Photophysics of thermally activated delayed fluorescence molecules. *Methods Appl. Fluoresc.* **5**, 012001 (2017).
4. Baleizão, C. & Berberan-Santos, M. N. Thermally activated delayed fluorescence as a cycling process between excited singlet and triplet states: Application to the fullerenes. *J. Chem. Phys.* **126**, 204510 (2007).
5. Etinski, M., Tatchen, J. & Marian, C. M. Time-dependent approaches for the calculation of intersystem crossing rates. *J. Chem. Phys.* **134**, 154105 (2011).
6. Marian, C. M., Heil, A. & Kleinschmidt, M. The DFT/MRCI method. *WIREs Comput. Mol. Sci.* **9**, e1394 (2019).
7. Grimme, S. & Waletzke, M. A combination of Kohn–Sham density functional theory and multi-reference configuration interaction methods. *J. Chem. Phys.* **111**, 5645–5655 (1999).
8. Becke, A. D. Density-functional exchange-energy approximation with correct asymptotic behavior. *Phys. Rev. A* **38**, 3098–3100 (1988).
9. Becke, A. D. A new mixing of Hartree–Fock and local density-functional theories. *J. Chem. Phys.* **98**, 1372–1377 (1993).
10. Becke, A. D. Density-functional thermochemistry. III. The role of exact exchange. *J. Chem. Phys.* **98**, 5648–5652 (1993).
11. Grimme, S., Ehrlich, S. & Goerigk, L. Effect of the damping function in dispersion corrected density functional theory. *J. Comput. Chem.* **32**, 1456–1465 (2011).
12. Lee, C., Yang, W. & Parr, R. G. Development of the Colle-Salvetti correlation-energy formula into a functional of the electron density. *Phys. Rev. B* **37**, 785–789 (1988).
13. Grimme, S., Antony, J., Ehrlich, S. & Krieg, H. A consistent and accurate ab initio parametrization of density functional dispersion correction (DFT-D) for the 94 elements H-Pu. *J. Chem. Phys.* **132**, 154104 (2010).
14. Stephens, P. J., Devlin, F. J., Chabalowski, C. F. & Frisch, M. J. Ab Initio Calculation of Vibrational Absorption and Circular Dichroism Spectra Using Density Functional Force Fields. *J. Phys. Chem.* **98**, 11623–11627 (1994).
15. Tatchen, J., Gilka, N. & Marian, C. M. Intersystem crossing driven by vibronic spin–orbit coupling: a case study on psoralen. *Phys. Chem. Chem. Phys.* **9**, 5209 (2007).
16. Heil, A., Kleinschmidt, M. & Marian, C. M. On the performance of DFT/MRCI Hamiltonians for electronic excitations in transition metal complexes: The role of the damping function. *J. Chem. Phys.* **149**, (2018).
17. Schäfer, A., Huber, C. & Ahlrichs, R. Fully optimized contracted Gaussian basis sets of triple zeta valence quality for atoms Li to Kr. *J. Chem. Phys.* **100**, 5829–5835 (1994).
18. Schreiber, M., Silva-Junior, M. R., Sauer, S. P. A. & Thiel, W. Benchmarks for electronically excited states: CASPT2, CC2, CCSD, and CC3. *J. Chem. Phys.* **128**, 134110 (2008).
19. Silva-Junior, M. R., Schreiber, M., Sauer, S. P. A. & Thiel, W. Benchmarks for electronically excited states: Time-dependent density functional theory and density functional theory based multireference configuration interaction. *J. Chem. Phys.* **129**, 104103 (2008).
20. Furche, F. *et al*. Turbomole. *WIREs Comput. Mol. Sci.* **4**, 91–100 (2014).
21. Slavíček, P. & Martínez, T. J. *Ab initio* floating occupation molecular orbital-complete active space configuration interaction: An efficient approximation to CASSCF. *J. Chem. Phys.* **132**, 234102 (2010).
22. Schäfer, A., Horn, H. & Ahlrichs, R. Fully optimized contracted Gaussian basis sets for atoms Li to Kr. *J. Chem. Phys.* **97**, 2571–2577 (1992).
23. Weigend, F. & Ahlrichs, R. Balanced basis sets of split valence, triple zeta valence and quadruple zeta valence quality for H to Rn: Design and assessment of accuracy. *Phys. Chem. Chem. Phys.* **7**, 3297 (2005).
24. Hohenstein, E. G. *et al*. Analytic first derivatives of floating occupation molecular orbital-complete active space configuration interaction on graphical processing units. *J. Chem. Phys.*

143, 014111 (2015).
25. Hohenstein, E. G. Analytic formulation of derivative coupling vectors for complete active space configuration interaction wavefunctions with floating occupation molecular orbitals. *J. Chem. Phys.* **145**, 174110 (2016).
26. Seritan, S. *et al.* TeraChem: A graphical processing unit-accelerated electronic structure package for large-scale ab initio molecular dynamics. *Wiley Interdisciplinary Reviews: Computational Molecular Science* vol. 11 e1494 (2021).
27. Ufimtsev, I. S. & Martínez, T. J. Quantum chemistry on graphical processing units. 1. strategies for two-electron integral evaluation. *J. Chem. Theory Comput.* **4**, 222–231 (2008).
28. Ufimtsev, I. S. & Martinez, T. J. Quantum chemistry on graphical processing units. 3. Analytical energy gradients, geometry optimization, and first principles molecular dynamics. *J. Chem. Theory Comput.* **5**, 2619–2628 (2009).
29. Fales, B. S. & Levine, B. G. Nanoscale Multireference Quantum Chemistry: Full Configuration Interaction on Graphical Processing Units. *J. Chem. Theory Comput.* **11**, 4708–4716 (2015).
30. Fales, B. S. & Martínez, T. J. Fast transformations between configuration state function and Slater determinant bases for direct configuration interaction. *J. Chem. Phys.* **152**, 164111 (2020).
31. Ben-Nun, M. & MartíNez, T. J. Ab initio quantum molecular dynamics. *Adv. Chem. Phys.* **121**, 439–512 (2002).
32. Ben-Nun, M. & Martínez, T. J. Nonadiabatic molecular dynamics: Validation of the multiple spawning method for a multidimensional problem. *J. Chem. Phys.* **108**, 7244–7257 (1998).
33. Ben-Nun, M., Quenneville, J. & Martínez, T. J. Ab initio multiple spawning: Photochemistry from first principles quantum molecular dynamics. *J. Phys. Chem. A* **104**, 5172–5175 (2000).
34. Adamo, C. & Barone, V. Toward reliable density functional methods without adjustable parameters: The PBE0 model. *J. Chem. Phys.* **110**, 6158–6170 (1999).
35. Perdew, J. P., Burke, K. & Ernzerhof, M. Generalized Gradient Approximation Made Simple. *Phys. Rev. Lett.* **77**, 3865–3868 (1996).
36. Wigner, E. On the Quantum Correction For Thermodynamic Equilibrium. *Phys. Rev.* **40**, 749–759 (1932).
37. Ieuji, R., Goushi, K. & Adachi, C. Triplet–triplet upconversion enhanced by spin–orbit coupling in organic light-emitting diodes. *Nat. Commun.* **10**, 5283 (2019).
